# Sulfide-oxidizing potential and hypersalinity tolerance strategies in salt-crust covered coastal microbial mats

**DOI:** 10.1093/ismejo/wrag166

**Published:** 2026-06-26

**Authors:** Dimitri V Meier, Andreas Greve, Dirk de Beer, Raeid M M Abed, Dagmar Woebken

**Affiliations:** Chair of Ecological Microbiology, Bayreuth Center of Ecology and Environmental Research (BayCEER), University of Bayreuth, Dr.-Hans-Frisch-Straße 1-3, D-95448 Bayreuth, Germany; Department of Microbiology and Ecosystem Science, Centre for Microbiology and Environmental Systems Science, University of Vienna, Djerassiplatz 1, A-1030 Vienna, Austria; Microsensor Group, Max Planck Institute for Marine Microbiology, Celsiusstrasse 1, D-28359, Bremen, Germany; Microsensor Group, Max Planck Institute for Marine Microbiology, Celsiusstrasse 1, D-28359, Bremen, Germany; Biology Department, College of Science, Sultan Qaboos University, P. O. Box: 36, PC 123 Al Khoud, Sultanate of Oman; Department of Microbiology and Ecosystem Science, Centre for Microbiology and Environmental Systems Science, University of Vienna, Djerassiplatz 1, A-1030 Vienna, Austria

**Keywords:** hypersalinity, sulfide oxidation, oxygen stress, desiccation tolerance, metagenomics, comparative genomics, cyanobacteria

## Abstract

Hypersaline microbial mats are dense microbial ecosystems capable of performing nearly complete element cycling under harsh conditions including near-saturation salinity. Our previous study of salt-crust-covered microbial mats showed that oxygenic photosynthesis was inhibited at salt saturation, while phototrophic sulfide oxidation persisted despite well-known sulfide-oxidizing taxa being undetectable. In this study, we analyzed metagenome-assembled genomes (MAGs) from the same mats to identify sulfide-oxidizing taxa and adaptations enabling oxygenic phototrophs to survive salt saturation. We extended the dataset by including morphologically identical mats exposed to lower salinity regimes to identify metabolic capabilities specifically selected for by saturation-level salinity. The phototrophic sulfide oxidation capability was found in nearly all cyanobacterial MAGs, in some *Chloroflexota*, and in abundant *Rhodovibrio* populations previously not known to oxidize sulfide. Furthermore, we found clear indications of *Haloarchaea*-like potassium-based osmoregulation in *Bradymonadaceae* (*Myxococcota*) adding another taxon to the few known potassium-accumulating bacteria. Despite lower oxygen concentrations, salt-crust-covered mats showed smaller proportions of fermenters and higher proportions of aerobic microorganisms than lower-salinity mats. We compared the genetic signatures of hypersalinity and desiccation tolerance in cyanobacterial MAGs from this study to genomes from desiccation-prone environments such as desert soils and small freshwater streams. Genomes of hyperhalophilic cyanobacteria were characterized by lack of certain potassium transporters and catalase genes and presence of additional osmolyte transporter subunits and sulfide-oxidation genes. We hypothesize that during salt saturation the oxidative stress for mat dwelling cyanobacteria is lowered, while the ability to oxidize sulfide provides them with energy when oxygenic photosynthesis is inhibited.

## Introduction

Microbial mats are nearly self-sustained microbial ecosystems with complete cycling of elements that can be found on the sediment surface of coastal areas [1]. Primary production of organic carbon and nitrogen is performed by photoautotrophic cyanobacteria and algae, whereas the generated organic matter is recycled by aerobic heterotrophs in the upper layers, and by denitrifiers, sulfate reducers, and fermenters in the deeper mat layers [2]. Sulfide produced by sulfate reduction, in turn, is oxidized by chemolithotrophic and phototrophic sulfide oxidizers in layers above [2]. Such coastal microbial mats also exist at low latitudes, on coastal flooding plains with high water evaporation and salt precipitation, also known as sabkhas [3–5]. With their diversity of microbial taxa, as well as high biomass, hypersaline microbial mats are a suitable system to study microbial strategies of osmoregulation and the effects of hypersalinity on different microbial metabolisms. Hypersalinity stress has often been compared to desiccation stress because, in both cases, water is being withdrawn from the cells [6, 7], and some of the cyanobacteria found in the microbial mats are taxonomically related to cyanobacteria found in desert soil crusts (e.g. family *Coleofasciculaceae* [8]). This existence of related microorganisms with the same general metabolism (photoautotrophy) and similar lifestyle (surface habitat exposed to heat and high solar irradiation) in a different water-limited environment presents an opportunity to identify potential differences in genetic adaptations to hypersalinity and extreme desiccation stress.

The main challenge faced by the microorganisms inhabiting such hypersaline microbial mats is the low availability of water due to hyperosmotic conditions imposed by high concentrations of NaCl. Two fundamentally different strategies to keep water inside the cell are known in the microbial world. The first is called the “salt-out” strategy and relies on production of small organic molecules, called osmolytes, that do not interfere with intracellular metabolism yet establish a strong osmotic pull that counters the high extracellular concentrations of ions [9, 10]. The other strategy is called “salt-in” as it is based on tolerance of high concentrations of ions inside the cell [9]. The salt-in strategy is mainly known from halophilic archaea and only a few bacterial exceptions such as *Salinibacter ruber* [11]. The distributions of these osmoregulation strategies among the largely uncultured members of hyperhalophilic microbial communities remain underexplored. Whether a microorganism can “afford” the energetic costs of countering hyperosmotic stress, stay active, and grow at high NaCl concentrations depends on its osmoregulation strategy, as well as on the energy and carbon sources it uses [12]. Hence, different levels of salinity should select for different metabolic traits. Although gradual shifts of microbial mat community composition with salinity gradient are documented by marker gene studies [13–16], the functional underpinnings of these shifts are not fully understood due to lack of representative cultures or genomes of many affected taxa. Few metagenomic studies of hypersaline microbial mats exist [17–22], performed on lower-salinity mats (<20% NaCl, mostly <10% NaCl) and not focusing on the impact of different salinity levels on the functional makeup of the community. An exception is a recent salinity manipulation study performed on salt-saturated microbial mats from a solar saltern on Mallorca Island, Spain, where different amplitudes of salinity variation have been shown to select different species within the abundant bacterial and archaeal genera over the course of over 800 days [23].

**Figure 1:**
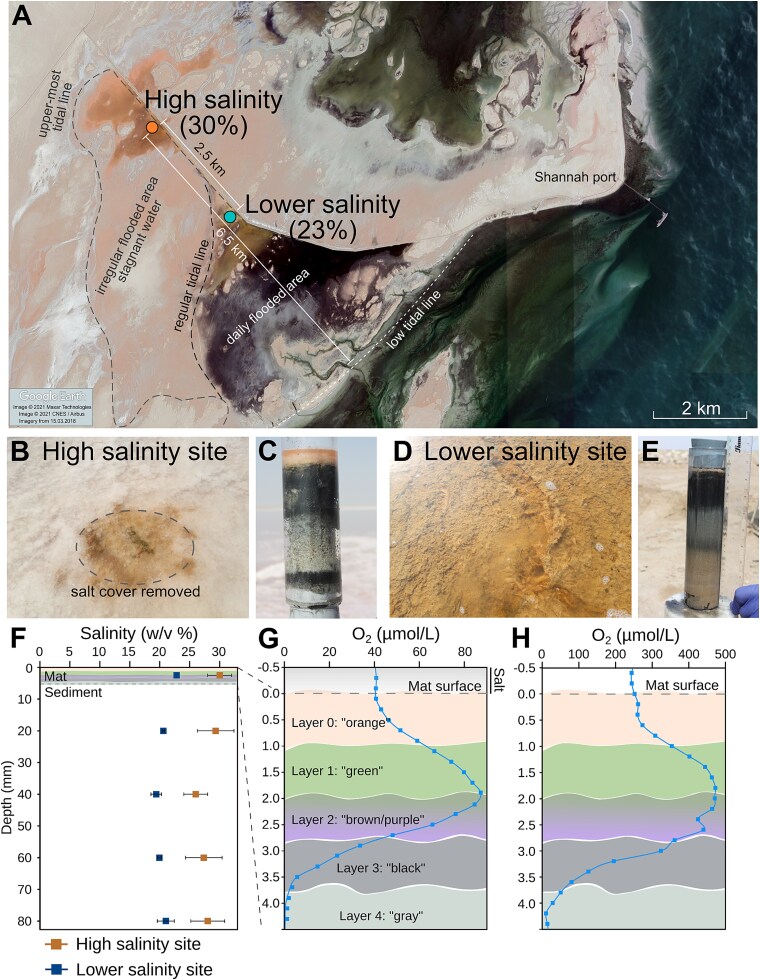
Location, surface appearance, *in situ* salinity, and oxygen profiles of the sampled microbial mats. (A) Locations of the two mat sampling sites within the Shannah sabkha with indicated extensions of regular diurnal tides and rare strong tides. Two filled circles mark the sampling sites with the salinity of mat pore water at the time of sampling indicated in % NaCl (w/v) under the label. Satellite image obtained from Google Earth, Image © 2021 Maxar Technologies, Image © 2021 CNES/Airbus, Imagery from 15.03.2018 **(**B–E) Appearance of the mat surface and sediment columns below the mats at HS (B, C) and LS (D, E) site. (F) Porewater salinity in the mats and underlying sediments of the two sampling sites measured in 3 independent cores per site. Average values and standard deviations are shown. (G, H) O_2_ concentration profiles measured at HS site at 11:36 at sunlight intensity of ~1760 μmol · m^−2^ · s^−1^ (G) and at LS site at 13:47 at sunlight intensity of ~1630 μmol · m^−2^ · s^−1^ (H). Approximate distribution of mat layers is indicated behind the profiles. Light intensities and microsensor profiles were measured as described previously [24].

At the sabkha near Shannah port in the Sultanate of Oman (Fig. 1), extensive microbial mats exist up to saturation-level salinities, leading to salt crust precipitation at the upper coast line [15, 24]. Although salinity shift experiments on these mats were previously performed to assess their short-term effect on microbial activities [24], the longer-term effect on mat-forming microbial communities in terms of taxa and genetic potential selected for by salt saturation conditions have not yet been investigated. We hypothesized that the composition of microbial communities in different parts of the salinity gradient could be the result of two largely independent processes: (i) elimination of taxa that are unable to survive at salt saturation and (ii) enrichment of taxa that are specifically adapted to grow better at salt saturation.

Beyond the osmotic stress itself, salt saturation has other effects on microbial activity in the mats. Our previous microsensor-based study of microbial processes in these mats showed that oxygenic photosynthesis performed by cyanobacteria was minimal at 30% NaCl (w/v) and was completely paused at salt oversaturation (40% w/v NaCl), rendering the mat anoxic below 2 mm depth [24]. In contrast, anoxygenic photosynthesis via phototrophic sulfide oxidation was maintained at all conditions [24]. However, in our 16S rRNA gene sequence data, known sulfur-oxidizing microorganisms such as *Halorhodospira* [25, 26], *Halochromatium* [27–29], *Thiohalocapsa* [18], or *Campylobacterales* [30] were largely absent (<1% relative abundance) [24]. Furthermore, the mats contained microscopically visible filamentous cyanobacteria [24], traditionally considered to be limited to salinities below 15% NaCl [31]. These observations raised the questions of who are the key sulfur oxidizers in this ecosystem, and how do the cyanobacteria endure the extended periods where oxygenic photosynthesis is inhibited? Some cyanobacteria are known to form resting stages called akinetes when conditions become unfavorable [32]. However, the taxa identified in these mats are not known to form such specialized dormant cells [33–35]. We hypothesized that the cyanobacteria inhabiting these mats are capable of sulfur oxidation, as previously observed in sulfidic springs [36–38] and cultures [39–42]. Alternatively, other bacterial taxa, not yet known to possess this capability, could be responsible for phototrophic sulfur oxidation. Earlier work suggested that *Candidatus* Chlorothrix related microorganisms (*Chloroflexota*) might be major sulfur oxidizers in these mats [24]. *Candidatus* Chlorothrix has been previously reported to grow autotrophically while oxidizing sulfide, albeit not in pure culture [43]. However, no (meta-)genome of the enrichment culture was published and it is still unknown whether *Ca*. Chlorothrix encodes sulfur-oxidation genes.

In order to investigate the adaptations of mat microorganisms to salt saturation conditions, we analyzed metagenomes of microbial mats from the Shannah sabkha (Fig. 1). More specifically, we obtained depth-resolved samples from the salt-crust covered part of the lagoon (Fig. 1B and C), same sampling material as analyzed previously [24], further referred to as a “high-salinity” or “HS” site, and from morphologically identical mats from the lagoon part devoid of salt precipitates and inundated by less saline water (overlaying water 18%, pore water in the mat: 23% NaCl w/v, Fig. 1D and E), further referred to as a “lower salinity” or “LS” site. We complemented the metagenomic data with previously published cell quantification and *in situ* oxygen profile data from the HS site [24] and additional cell count and oxygen data from the LS site (Fig. 1F). As a result, we identified microbial metabolisms preferentially selected by saturation-level salinity, revealed the key S-oxidizing microorganisms and pathways, and elucidated metabolisms helping cyanobacteria to survive oxygenic photosynthesis-inhibited conditions. Furthermore, we identified clear genetic adaptations of halophilic cyanobacteria to extreme osmotic stress.

## Materials and methods

### Site description and sampling

Microbial mats were sampled at a coastal sabkha near the port of Shannah, Sultanate of Oman. The sabkha is created by tides bringing seawater into a large shallow tidal flat (Fig. 1) that subsequently evaporates, leading to increased salt concentrations up to salt saturation and formation of a salt crust in some areas. Microbial mats observed throughout the tidal flat were first described and sampled for marker-gene-based diversity analysis in 2014 [15]. The samples used in this study were taken at two sites with different salinity regimes yet visually identical microbial mats (Fig. 1) in February 2018. Microbial mats at the High Salinity site (20.7610, 58.6478) were previously described with respect to microbial process rates at different salinities and distribution of cells, pigments, and microbial taxa across distinct mat layers [24]. At the end of the sampling campaign, we identified a second location with visually identical microbial mats yet not covered by a crust of salt, here referred to as the Lower Salinity site (20.7452, 58.6625). In addition to previously published biogeochemical data from the HS site [24], here, we report field salinity (refractometer measurements) and oxygen microprofiles, as well as cell densities for microbial mats from the LS site. In this study, we sequenced metagenomes from the same samples as described previously [24] and additional samples from microbial mats at the LS site.

At both the HS site and the LS site, three independent patches of microbial mats were collected ca. 1 m away from the microsensor measurement site. At the HS site, salt crust was removed from the mat surface and the surface was flushed with deionized water. For each mat patch, five ca. 1 mm–thick layers were identified according to their color—orange, green, brown, black, and gray—and separated with a scalpel. The material from each layer was collected into 2 ml microcentrifuge tubes and immediately transferred to dry ice. Upon return from the field, samples were stored at −20°C. A fraction of the material (~500 mg) was preserved for fluorescence microscopy analysis by fixation with 4% paraformaldehyde (PFA) in 1× phosphate-buffered saline (PBS) (pH = 7.6) for 1.5 h at room temperature, then washed twice with 1× PBS and stored in an ethanol/PBS mixture (1:1) at −20°C until further analysis.

### Field oxygen and salinity measurements

Field oxygen microprofiles were measured identical to the profiles described previously [24] using microsensors and procedures described elsewhere [44]. The mat surface was defined as zero depth. Microsensor oxygen measurements were performed on undisturbed microbial mats prior to any other sampling of mat material.

Within 1 m radius from the microsensor measurement point, three independent sediment cores were taken with plexiglass core liners with pre-drilled holes for Rhizon (Rhizosphere Research Products, Wageningen, The Netherlands) insertion. Pore water was extracted with Rhizons in 2 cm intervals, and salinity was measured by refractometer after a 1:10 dilution with deionized water.

### Microscopy

For microscopic quantification of cell densities, cells were detached from particles and biofilm matrices by seven rounds of sonication of 80–200 mg of PFA-fixed sample as described previously [24], and the combined supernatant was filtered on 0.2 μm pore-size polycarbonate filters (Sartorius, Göttingen, Germany), 30 μl per filter. The 4′,6-diamidine-2′-phenylindole dihydrochloride (DAPI)–stained cells were quantified with a fluorescent microscope (10 images per sample). For filamentous microorganisms, individual cells in the filament were counted, not filaments as a whole. Cell numbers were back calculated to the wet weight of the original sample.

### DNA extraction

DNA extraction was performed exactly as described previously [24, 45]. Briefly, ca. 400 mg of sample were washed with 1 × PBS (pH = 7.4) twice before extracting DNA with a phenol:chloroform-based protocol including 3 × 30 s of bead beating at 6.5 m s^−1^ in a FastPrep-24 (MP Biomedicals, Irvine, CA, USA), complexation of polymeric organic substances with 5% CTAB, standard chloroform:isoamylalcohol (24:1) washing, and DNA precipitation with 20% PEG 8000 in 2.5 M NaCl. Detailed protocol steps and solution recipes can be found under the following link: https://doi.org/gf6mq3. The extracted DNA was sent to the Joint Genome Institute of the Department of Energy of the USA for sequencing on a NovaSeq 6000 System (Illumina, San Diego, CA, USA) in 2 × 150 bp paired end mode, metagenome assembly, and functional annotation.

### Metagenome sequencing, assembly, and annotation

Out of 30 samples, one library construction has failed (LS site Mat VIII Layer 4), resulting in 29 successfully sequenced metagenomes. The generated short reads (Tab. S1) were analyzed with the standard Joint Genome Institute (JGI) pipeline. Briefly, reads were quality-filtered using the BBtools command bbcms v.38.44 [46] with the options “*mincount = 2 highcountfraction = 0.6*” and assembled using SPAdes v.3.13.0 [47] with command line options “*--only-assembler -k 33,55,77,99,127 –meta*.” Genes on the contigs were annotated with the Integrated Microbial Genomes (IMG) Annotation Pipeline v.5.0.14 [48]. Only contigs longer than 1000 bp were used for all analyses.

Genes encoding key metabolic functions were searched in the annotation table with Structured Query Language (SQL) search queries combining multiple search criteria, referring to different annotation sources. Used SQL queries are deposited on Figshare (“09_Function_search.sql” under DOI:10.6084/m9.figshare.28494914, https://figshare.com/ndownloader/files/63546522). Full multi-source annotations of all searched genes can be found on Figshare in the “Annotations_of_searched_functions.tar.gz” archive under DOI:10.6084/m9.figshare.28495778. 

### Metagenome binning

Metagenome binning was performed for the assemblies of 10 deeply sequenced metagenomes (Mat V and Mat IX, Tab. S1), five mat layers from each site. The exact commands can be found in bash scripts deposited on Figshare under DOI:10.6084/m9.figshare.28494914 and numbered in the order of analysis steps. Read coverage information was obtained by mapping the filtered reads from all 29 samples to each of the 10 assemblies using BBmap v. 38.49 [46] with the parameters “*minid = 97 idfilter = 95*.” Binning was performed for each of the 10 assemblies using MetaBAT2 v. 2.12.1 [49], MaxBin v.2.2.4 [50], and CONCOCT v. 0.4.1 [51] with subsequent summarizing of binning results by DASTool v. 1.1.0 [52] with a score threshold of 0.2. Genomic bins generated for each of the assemblies were dereplicated with dRep v.1.4.3 [53] resulting in one nonredundant set of 487 bins for the whole dataset. Filtered reads were mapped once again to the final bin set to create coverage profiles for the dereplicated bin collection.

An Anvi’O v. 6.1 [54] database was created using the contigs of the dereplicated bin collection and their coverage profiles. Bin quality was assessed using CheckM v. 1.1.3 [55]. Contamination was calculated as the percentage of lineage-specific single-copy genes present in two divergent copies (CheckM reports contamination in reference to all duplicated genes, not all single-copy genes). Genomic bins with contamination values >10% were inspected in Anvi’O and contigs with divergent tetranucleotide profiles and coverage profiles were removed from the bins in an attempt to reduce the contamination. Furthermore, all SSU and LSU rRNA genes identified by Anvi’O were exported and classified using SINA v 1.2.11 [56] and the SILVA 138.1 database [57]. The bins were taxonomically classified using GTDB-Tk v2.6.1 [58] and the GTDB database v.226 [59]. Contigs with rRNA sequences classified differently than the bin were removed from the bins.

After filtering based on completeness (>50%) and contamination (<10%) values, 390 bins remained representing 40% ± 8% of the unassembled reads in individual samples (Tab S1). The bins had an average completeness 74% ± 14% of and a contamination of 3% ± 2%. This set of metagenome-assembled genomes (MAGs) was used for all analyses of functional potential in this study. MAGs were assigned general metabolic categories based on presence or absence of indicator genes (Tab. S2). The final MAGs are deposited as FASTA files (contig sequences and protein sequences) on Figshare (DOI:10.6084/m9.figshare.28494731).

### Sulfur-oxidation gene analysis

MAGs were searched for the following genes indicative of the ability to oxidize reduced sulfur species: sulfide:quinone oxidoreductase (Sqr), flavocytochrome *c* sulfide dehydrogenase (Fcc), genes encoding the subunits of the SOX multi-enzyme system: SoxA, SoxX, SoxB, SoxY, SoxZ, and SoxC. Knowing that the Sqr can also have a purely detoxifying function, a brief phylogenetic analysis was conducted (Fig. S1) in order to attribute the encoded Sqr proteins to the different enzyme types with known function according to Marcia *et al*. [60]. Sqr amino acid sequences used for phylogenetic distinction of the different Sqr types [60] were downloaded either from Uniprot or the National Center for Biotechnology Information (NCBI) protein database, aligned together with Sqr sequences from the MAGs using MAFFT v. 7.490 [61] in L-INS-I mode, and used for phylogenetic tree calculations with FastTree v. 2.1.11 [62] based on alignment positions conserved in at least 25% of the sequences. Tree calculations were performed with the Le-Gascuel substitution model [63], the BioNJ [64] starting tree, and gamma likelihood optimization. Tree visualization and exploration was done in ARB v. 7.0 [65]. The Arb database containing amino acid sequence alignments and the phylogenetic tree, as well as the exported tree in Newick format, can be found on Figshare under DOI:10.6084/m9.figshare.28494914 in the “Analysis_and_plotting” folder. Only Sqr types I, IV, and VI were considered indicative of energy generating sulfide oxidation, as enzymes of these types were previously shown to enable growth on sulfide as sole electron donor [60]. MAGs were considered to represent potential sulfur oxidizers if they contained either genes for Sqr type I, IV, or VI, or more than one gene-encoding subunits of the SOX system or the Fcc. Genes indicative of the heterodisulfide reductase (Hdr)–like sulfur-oxidizing complex and associated sulfur carrier proteins were searched based on previous descriptions of the pathway [66, 67]. We especially oriented our search based on the EggNOG orthologous groups and Pfam domains cross-referenced in the Uniprot database for hdr-like, lbpA, and lbpA maturation genes identified in *Chloroflexota* bacterium *Thermomicrobium roseum* [66], to test if the phototropic *Chloroflexota* found in the mats could use the Hdr-based pathway for sulfur oxidation. However, presence of putative Hdr-based sulfur oxidation genes (see File S1) was not considered as a definitive indicator of sulfur-oxidizing potential, as the pathway was discovered very recently and it is not yet clear what its distinctive genetic markers are and how it can be differentiated from other functions of Hdr.

### Analysis of isoelectric point of encoded proteins and osmoregulation-related genes

The isoelectric points of proteins encoded by each MAG were calculated using the iep tool from the EMBOSS package v. 6.6.0 [68]. Homologs of *Salinibacter* salt-in genes [11] were used as indicators of salt-in osmoregulation strategy, whereas genes involved in betaine, ectoine, and trehalose synthesis were used as indicators of compatible solute-based osmoregulation. Homologs of *S. ruber* potassium transporters TrkA and TrkH were downloaded from the Uniprot database, aligned with MAFFT v. 7.490 [61] in L-INS-I mode, together with TrkA and TrkH protein sequences from the MAGs, and used for phylogenetic tree calculations with FastTree v. 2.1.11 [62] based on alignment positions conserved in at least 25% of the sequences. Tree calculations were performed with the Le-Gascuel substitution model [63], BioNJ [64] starting tree, and gamma likelihood optimization. Tree visualization and exploration were done in ARB v. 7.0 [65].

A separate subtree calculation was performed for the cluster largely containing TrkA sequences of extremophile (halophile or thermophile) microorganisms. The main aim of phylogenetic tree calculations was to determine the relationship of sequences from *Bradymonadaceae* MAGs to sequences of *Archaea* and *S. ruber*. The overall topology of all TrkA and TrkH proteins was not evaluated. Calculation of trees only based on reference sequences with subsequent insertion of MAG sequences was also performed, as well as tree calculations without the positional conservation filter. The cluster of extremophile sequences separate from the general bacterial cluster and the placement of *S. ruber* sequences with the halophilic Archaea sequences, as well as clustering of certain *Bradymonadaceae* TrkA sequences as a sister clade to *Salinibacter* sequences, were reproducible in all calculations.

### Comparison of cyanobacterial genomes

Amino acid alignments and phylogenetic trees produced by GTDB-Tk (GTDB tree of bacterial genomes with inserted MAGs) were loaded into ARB [69], together with GTDB metadata providing information on NCBI BioSample entries associated with the genomes and isolation sources of the organisms. Sequences of cyanobacteria were used for a *de novo* tree calculation based on alignment positions conserved in at least 25% of the sequences using Fast Tree v. 2.1.11 [62] with “*-bionj -lg -gamma*” parameters.

Based on the *de novo* calculated cyanobacteria phylogenetic tree, related genomes from terrestrial, freshwater, marine, and hypersaline/salt saturated environments were selected for comparisons. The analysis was limited to the order *Cyanobacteriales*, excluding two *Phormidesmidales* MAGs, to avoid comparing too distant genomes. Protein sequences of the genomes were downloaded from NCBI based on the accession numbers in the tree. If protein sequences for a genome were not readily available on NCBI, genes were predicted and translated using Prodigal v. 2.6.2 [70]. The proteins encoded by each genome were assigned to EggNOG orthologous groups using EggNOG mapper v. 2.1.4 [71], and a table of presence–absence of orthologous groups in genomes was generated using bash command line and python. Distribution of EggNOG orthologous groups related to reactive oxygen species defense and osmoregulation across genomes from different habitats was evaluated.

### Analysis of taxa and metabolism distribution between high-salinity site and lower-salinity site

Quality-filtered metagenomic reads generated by the JGI were mapped to the SILVA SSU138.1 database using PhyloFlash v. 3.4 [72], and taxa recruiting three and more reads were considered present and were quantified based on read mappings. Eukaryotic sequences (including chloroplast and mitochondrial rRNA sequences) were removed from the mapping counts table and analyzed separately. Diversity indices (Shannon, Inverse Simpson) were calculated with the “diversity” function of the vegan R package v. 2.6-4 [73]. Species richness (number of all observed taxa in a sample) was calculated with the “specnumber” function in vegan v. 2.6-4 [73]. Differential abundance of microbial taxa (at the genus level) was tested using DeSeq2 (Wald test). Taxa that significantly contribute to community differences between HS site and LS site samples were identified using Similarity Percentage Analysis (SIMPER) as implemented [73]. Furthermore, the Wilcoxon rank-sum test in R package vegan was applied to estimated absolute abundance data of genera generated by multiplying relative abundances by microscopic cell counts per gram of microbial mat. Because the absolute count data varies by orders of magnitude between upper and lower mat layers, the test between the HS site and the LS site was run separately for “top” mat layers (0–2) and “bottom” mat layers (3 & 4). Taxa showing an adjusted *P*-value below .05 in at least one of the tests and a Log2-fold change bigger than 1 in at least one of the comparisons (relative abundance, absolute abundance, or DeSeq2-corrected abundance) were considered differentially abundant. Taxa that did not show a Log2-fold change greater than one exhibited contradictory Log2-fold changes (e.g. between absolute abundance and DeSeq2-corrected abundances).

To test the differential abundance of functional categories only the Wilcoxon rank-sum test on estimated absolute abundance data was used as described above. Abundance of the functional category of organisms (defined based on criteria in Tab. S2) was calculated as a sum of abundances of MAGs encoding the genes indicative of the respective function (Tab. S2). Because the functional categories are not mutually exclusive and one MAG can simultaneously contribute to different functional categories (e.g. S-oxidation, O_2_-respiration, and NO_3_-reduction), no tests for compositional data like DeSeq2 can be applied.

## Results

### Identification of a lower-salinity site

Exploring the sabkha where we found the salt crust-covered mats (at HS site, 30% w/v NaCl, Fig. 1A–C) following the gradient described previously [15], we found a site with morphologically identical mats yet without visible salt precipitation and covered by at least ~10 cm of water throughout the tidal cycle (LS site, Fig. 1A, D, and  E). Overlaying water salinity at this site was determined to be 18% (w/v NaCl), while pore water salinity within the mat was 23% (w/v NaCl). Although the LS site is still hypersaline, the maximum salinity never reached salt saturation as in the case of HS site. Based on field microsensor measurements, the oxygen penetration depth (~4 mm) and the depth of the peak oxygen concentration (~2 mm) at the LS site were very similar to HS site mats (Fig. 1G and H); however, the net oxygen production was almost an order of magnitude higher (Fig. 1G and H). Mats from both sites were ~5 mm thick and consisted of five distinct layers, each ca. 1 mm thick on average. These layers were identified based on their dominant color, from top to bottom, as orange, green, dark brown with purple shimmer, black, and gray layers.

For comparing the microbial community composition and functional potential between the two sites, three random patches of mats at each site were sampled, each separated into five individual layers based on color and texture (30 samples in total).

### Community composition

**Figure 2:**
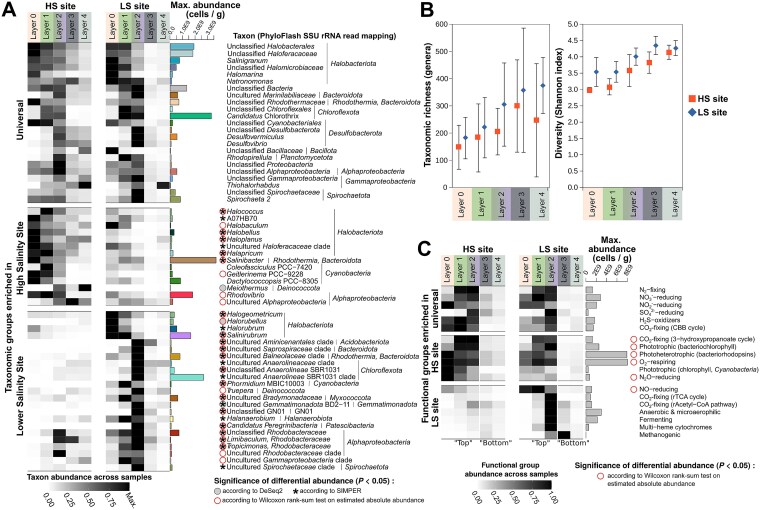
Overlaps and differences in microbial community composition between salt-covered Site 1 and salt-free Site 2. (A) Microbial genera distribution across mat layers at Site 1 and Site 2, as determined by sequence read mapping to the SILVA SSU database. For better visualization a cut-off of at least 1% relative abundance in any sample was applied for taxa with significant (*P* < .05, Log2-fold change >1) differential abundance between the sites according to DeSeq2 or Wilcoxon rank-sum test or with significant (*P* < .05) contribution to community differences between sites according to SIMPER. For taxa without statistically significant differences, the cut-off of at least 3% relative abundance was applied. Taxa that show a Log2fold change between −1 and 1 were considered as universal to both sites. (B) Richness (observed taxa) and diversity (Shannon Index) at genus level in different layers of the mats at Site 1 and 2. (C) Distribution of microbial metabolisms between two sites, based on genes encoded in reconstructed MAGs and MAG abundance across mat layers and sites.

To assess the effect of different salinities and tidal regimes on the microbial community composition, we compared the 16S rRNA gene sequences detected in the metagenomes of the two sites (Fig. 2A, Fig. S2). We performed similarity percentage (SIMPER) analysis to test the contribution of different microbial genera to the difference of community compositions between HS and LS sites and DeSeq2 analysis to identify significantly differentially abundant microbial genera (Fig. 2A).

Overall, the species richness and alpha diversity was slightly higher in each layer of LS than in HS (Fig. 2B). At both sites, the richness and diversity of the community composition was lower in the upper mat layers and increased with depth. Out of the 1225 genus-level taxa, 867 were detected in more than one sample. Although most genera were detected at both sites (642 genera), some low-abundant genera (⪯1% relative sequence abundance in any sample) were unique to HS site (73 genera present in more than one HS sample and absent from all LS samples). However, nearly twice as many genera were found to be unique to the LS site (152 genera present in more than one LS sample and absent from all HS samples). Only a few genera were significantly more abundant at the HS site (Wilcoxon rank-sum: 33 genera, SIMPER: 27 genera, DeSeq2: 6 genera), whereas many more genera were significantly more abundant at the LS site (Wilcoxon rank-sum: 99 genera, SIMPER: 454 genera, DeSeq2: 58 genera). In summary, the few cases of taxa unique or significantly more abundant at HS site were outweighed by cases of taxa whose abundance decreased or which completely disappeared in HS communities compared to LS. These patterns of presence-absence and differential abundance indicate that the process of elimination of taxa that are not adapted to salt saturation is the main driver of community differences between HS and LS. Although many taxa like *Ca.* Chlorothrix appeared at both sites in similar proportions (Fig. 2A, Fig. S2), others were either more abundant in HS or in LS mats (Fig. 2A). The HS site was characterized by higher relative abundance of various *Haloferacaceae Archaea* taxa, *Salinibacter*, *Rhodovibrio,* and *Geitlerinemma Cyanobacteria* (Fig. 2A, Fig. S2). The LS site contained significantly higher proportions of *Salinirubrum Archaea*, uncultured order SBR1031 (*Chloroflexota* class *Anaerolineae*), *Balneolaceae Rhodothermia*, *Halanaerobiota*, *Rhodobacteraceae Alphaproteobacteria*, and uncultured *Bradymonadaceae* (*Myxococcota*). Among the *Cyanobacteria*, a shift from *Rubidibacteraceae*, *Coleofasciculaceae,* and *Geitlerinemaceae* at HS site to *Oscillatoriaceae*, *Phormidesmiales*, and *Thermosynechococcaceae* at the LS site was observed, although only the changes in *Geitlerinema* and *Phormidium* were statistically significant. Taking advantage of metagenomic sequencing capturing all small subunit rRNA gene sequences, we took a separate look at eukaryotic photoautotrophs present in the samples. 18S rRNA gene sequences of diatoms were significantly more abundant in HS mats (Fig. 2A, Fig. S2), whereas sequences of the hypersaline alga *Dunaliella* were much more abundant in surface samples of LS site (0–1 mm) (Fig. 2A). However, they were also present in three samples from the HS site (one replicate each from 1, 3, and 4 mm depth).

### Functional potential

Metagenome binning resulted in 390 bins representing 40% ± 8% of the unassembled reads in individual samples (Tab. S1). The MAGs had an average completeness of 74% ± 14% and contamination of 3% ± 2%. According to GTDB-Tk classification, 368 MAGs represented previously unsequenced species; 147—a previously unsequenced genus, 37—an unsequenced family, and 4 MAGs belonged to previously unsequenced orders. However, many of identified taxa did not have any cultured or otherwise described and named representatives and were represented by metagenome-derived genomes. Out of 390 MAGs, 375 belonged to species that have no cultured strains, 284 MAGs belonged to genera with no cultured representatives, 170 MAGs were from entirely uncultured families, and 80 MAGs were from uncultured orders.

Based on the presence of indicator genes (Tab. S2), metabolisms were assigned to MAGs. Using the relative abundances of MAGs in the dataset scaled by cell numbers per gram sediment, we compared the differences in abundance of MAGs with certain metabolisms involved in energy generation and primary production between HS site and LS. Because one genome can simultaneously be counted into multiple functional groups, statistical tests comparing relative abundance or community composition could not be applied to data on distribution of functions. Instead, we used the Wilcoxon rank-sum test on estimated absolute abundances of each functional group (Fig. 2C). The comparisons between the HS site and the LS site were done separately for the “top” (Layer 0—Layer 2) and “bottom” parts (Layer 3 & 4) of the mat (Fig. 2C) to account for a strong drop in cell numbers below Layer 3. Many metabolisms were abundant at both sites with only slight differences (Log2fold difference < 1, Fig. 2C, indicated as “universal”). Regarding differentially abundant metabolisms, we found populations encoding bacteriochlorophyll synthesis and rhodopsins significantly more abundant (Wilcoxon rank-sum test *P* < .05) in the upper layers of HS site, as well as populations encoding potential for N_2_O reduction (Fig. 2C). It was striking that populations with micro-aerophilic and exclusively anaerobic metabolisms were much more abundant at the LS site, in the layer from 2–3 mm depth. However, due to appearing in only one layer, the number of samples (*n* = 3 vs *n* = 3) was not enough to determine statistical significance. These metabolic groups include microorganisms lacking cytochrome *c* oxidases (some are denitrifiers or microaerophiles relying on cytochrome *bd* oxidase only), strict fermenters, methanogens, microorganisms fixing CO_2_ via oxygen-sensitive reverse tricarboxylic acid (rTCA) cycle, or a strictly anaerobic reductive Acetyl-CoA pathway. Roughly half of microaerophilic (17 out of 30) and strictly denitrifying MAGs (22 out of 48) without cytochrome *c* genes contained catalase or peroxidase genes. Proportion of MAGs with catalases was lower among strict anaerobes (10 out of 61).

### Sulfur-oxidizing microorganisms

**Figure 3:**
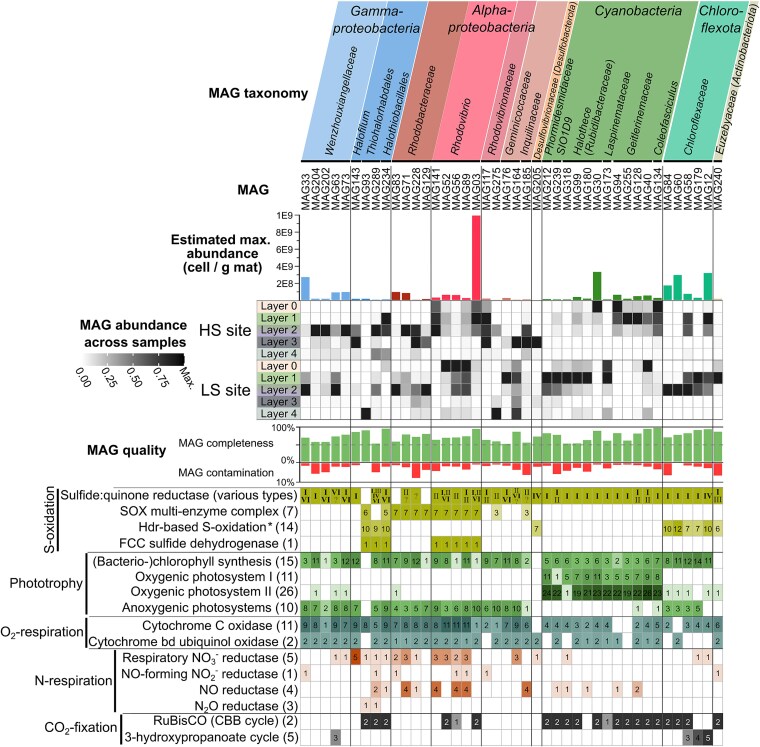
Taxonomic affiliation, abundance, and genetic potential for photo- and lithotrophic sulfide oxidation of MAGs representing potentially sulfur-oxidizing microbial populations. Population abundance was estimated by multiplying MAG relative abundance by microscopic cell counts. Heatmap is showing MAG abundance across samples relative to maximum abundance observed in any sample shown as a bar chart above the heat map. MAG quality is indicated as CheckM values for completeness and contamination. The table showing presence/absence of specific metabolisms indicates the number of genes encoding a pathway (e.g. chlorophyll synthesis) or subunits of an enzyme (e.g. SOX-multienzyme complex, 2 subunits of cytochrome *bd* ubiquinol oxidase). Numbers in brackets on the left indicate the maximum number of differently annotated genes encoding the pathway/enzyme found in a MAG. *Hdr-based S-oxidation pathway has only recently been discovered [66, 67], and its annotation in metagenomes is uncertain.

Unlike oxygenic photosynthesis, anoxygenic photosynthesis (phototrophic sulfide oxidation) was not inhibited completely by saturation-level salinity [24]. However, 16S rRNA gene sequences of microbial taxa typically associated with S-oxidation were only present at very low relative abundances in the mat communities [24]. Hence, we searched the obtained MAGs for sulfur-oxidation genes (encoding the sulfide–quinone reductase (Sqr), the subunits of the SOX complex, heterodisulfide reductase-like (Hdr-like) enzymes, and flavocytochrome *c* (FCC) and identified 42 potentially S-oxidizing populations (Fig. 3). Sqr can also serve sulfide detoxification. In total 158 out of 390 MAGs encoded some form of Sqr enzyme, including Type II Sqr also found in eukaryotes and yet uncharacterized archaeal and alphaproteobacterial Sqr clusters (Fig. S1). In the following, we are only focusing on Sqr types I, IV, and VI known to enable growth on sulfide as a sole electron donor [60, 74].

*Alphaproteobacteria* MAGs belonging to the genus *Rhodovibrio* and family *Rhodobacteraceae* had the most complete set of S-oxidation genes with Sqr and SOX encoding genes. *Rhodovibrio* MAGs additionally encoded the FCC sulfide dehydrogenase. MAGs of the family *Chloroflexaceae* related to *Ca.* Chlorothrix, three *Gammaproteobacteria* MAGs, one *Rhodobacteraceae* (*Alphaproteobacteria*) MAG, and the *Euzebyaceae* (*Actinobacteria*) MAG also encoded the Hdr-like enzymes, lipoate-binding proteins, and associated sulfur-carrier proteins involved in a recently described S-oxidation pathway found in some *Alphaproteobacteria* [66, 67]. The various *Cyanobacteria*, as well as *Wenzhouxiangellaceae* (Gammaproteobacteria), MAGs encoded only the sulfide–quinone reductase. Twenty-one out of 42 potentially S-oxidizing MAGs also encoded RuBisCO genes indicative of CO_2_-fixation via the Calvin–Benson–Bassham (CBB) cycle, including three *Chloroflexaceae* MAGs (Fig. 3). Additional two *Chloroflexaceae* MAGs contain genes (encoding biotin carboxylase, malonyl-CoA reductase, propionyl-CoA carboxylase, and propionyl-CoA synthase, File S1), suggesting possible CO_2_-fixation via the 3-hydroxypropanoate cycle (Fig. 3).

Almost all potential S-oxidizers encoded (bacterio-)chlorophyll synthesis pathways. The only exceptions were one *Thiohalorhabdus* MAG and one *Euzebyaceae* (*Actinobacteria*) MAG that lacked bacteriochlorophyll-synthesis genes and thus represented the only chemolithoautotrophic S-oxidizers. Four major groups of phototrophic sulfide oxidizers were *Chloroflexota*, *Cyanobacteria*, *Alpha*-, and *Gammaproteobacteria*. However, the possibility of light-independent sulfur oxidation with oxygen or nitrate as terminal electron acceptor also remained plausible as we identified genes for aerobic respiration in all potential S-oxidizer MAGs and genes for nitrate reduction in 18 MAGs.

### Phototrophy and primary production

Not only did 175 MAGs encode rhodopsin proton pumps, but also (bacterio-)chlorophyll synthesis genes were widely spread in the community (File S1). Seventy-one MAGs encoded more than three genes for the pathways of (bacterio-)chlorophyll or photosystem synthesis.

CO_2_-fixation genes, such as genes indicative of CBB, rTCA, reverse Acetyl-CoA (rAcetyl-CoA), and 3-hydroxypropanoate CO_2_-fixation pathways were present in 112 MAGs.

In total, 10% of all MAGs encoded nitrogen fixation genes (*nifH* and at least *nifD* or *nifK*). These genes were found not only in almost all *Cyanobacteria* but also in the MAGs of multiple other taxonomic groups, such as *Chloroflexaceae*, *Phycisphaerales Planctomycetota*, *Desulfobacterota*, *Inquilinaceae* and *Geminicoccaceae Alphaproteobacteria*, *Methanohalobium* archaeon, and others (File S1).

### Hypersalinity adaptations

#### Salt-in strategy in *Bradymonadaceae*

**Figure 4:**
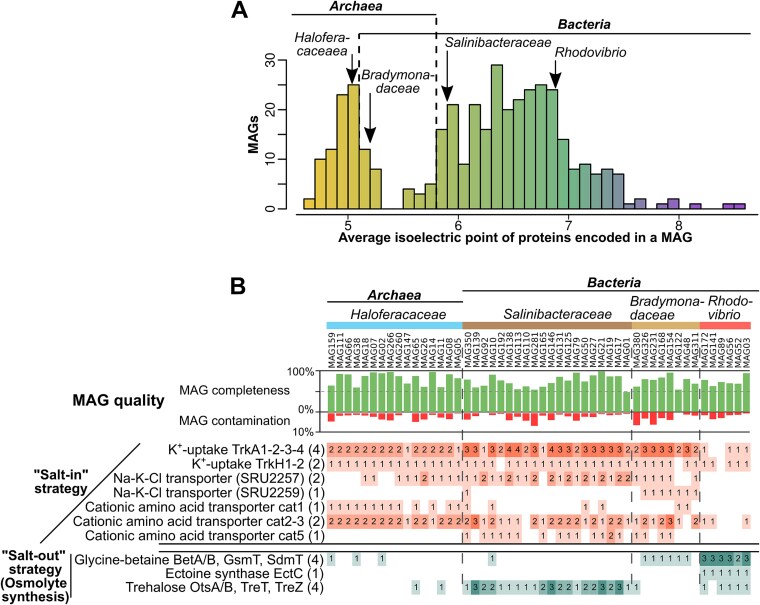
Indicators of different osmoregulation strategies of different microbial populations. (A) Distribution of proteome acidity (average isoelectric point of encoded proteins) among different MAGs. (B) Genes for potassium-based (salt-in) or organic osmolytes-based (salt-out) osmoregulation in *Bradymonadaceae* MAGs as well as example MAGs of microbial taxa with known osmoregulation strategies. Numbers in brackets on the left indicate the maximum number of differently annotated genes encoding the given enzyme/pathway found in a MAG (e.g. different genes of glycine-betaine synthesis pathways). The MAGs chosen as examples belong to halophilic archaea and one of the few bacterial taxa (*Salinibacteraceae*) known to use the salt-in strategy, as well as *Rhodovibrio* MAGs clearly encoding the osmolyte-based salt-out osmoregulation mechanisms as a control. The genetic potential and acidity of the proteome of *Bradymonadaceae* aligns well with the one of the salt-in microorganisms.

In order to assess the distribution of the salt-in and salt-out hypersalinity tolerance strategies in the microbial community, we calculated the average isoelectric points of encoded proteins for each MAG. An acidic proteome could be an indicator of the salt-in strategy. As expected, the encoded proteomes of the MAGs showed a binomial distribution (Fig. 4A) with most *Archaea* on the acidic end of the spectrum and most *Bacteria* on the more neutral or basic end. One exception were the MAGs of the *Bradymonadaceae* family that encoded very acidic proteomes comparable to the archaeal ones. The isoelectric point of the *Bradymonadaceae* proteins was even lower than that of *Salinibacter*, one of the few bacteria known to employ the salt-in strategy. To verify if *Bradymonadaceae* populations found in the mats indeed could be using the salt-in strategy, we screened their MAGs for genes indicative of this metabolism (mostly specific potassium transporters), homologous to the ones found in *Sainibacter* and *Haloarchaea*. Additionally, we searched for genes indicative of osmoregulation based on small organic molecules like betaine, ectoine, or trehalose. *Bradymonadaceae* MAGs encoded most of the potassium and cationic amino acid transporters considered to enable the salt-in hypersalinity tolerance strategy. In contrast, they encoded only one gene indicative of betaine synthesis and no genes for trehalose or ectoine synthesis. Phylogenetic analysis of the potassium transporter TrkA showed that some copies of the protein encoded in *Bradymonadaceae* MAGs were closely related to *Salinibacteraceae* transporters and fall into a sister cluster to the archaeal TrkA proteins which also include the *S. ruber* TrkA (Fig. S3). Other TrkA copies found in the same MAGs, however, showed no relation to halophilic organisms. For TrkH, no evidence of a close evolutionary relationship between *Salinibacteraecea* and *Bradymonadaceae* was found (Fig. S4).

#### Hypersalinity versus desiccation stress in *Cyanobacteria*

We performed comparative genomic analysis of cyanobacterial MAGs found in the mats together with their close relatives from other environments such as surface soils, and other hypersaline and salt-saturated environments, as well as marine and freshwater habitats (Tab. S3). Looking at genes related to osmotic stress and desiccation tolerance, we could identify clear differences between genomes from desiccation-prone habitats such as soils and small freshwater streams and genomes from habitats exposed to osmotic stress such as microbial mats.

**Figure 5:**
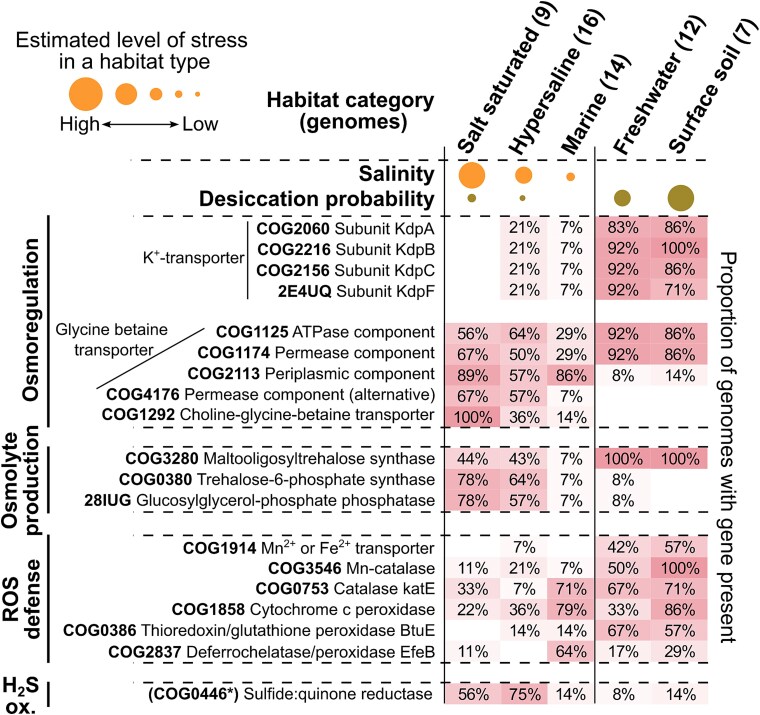
Differences in genetic potential of cyanobacterial genomes from different environments with respect to osmotic and xeric stress tolerance. The column headers indicate the source environments of cyanobacterial genomes and the number of genomes in a given category used for analysis. Most genomes in the “freshwater” category originate from small streams which are subject to water level fluctuation or seasonal drying up. The table shows the percentage of genomes in a certain category that encode a certain orthologous protein as annotated by EggNOG mapper. *although COG0446 contains proteins of diverse functions, the figure specifically summarizes only sulfide:quinone reductase encoding genes. Genomes used for the comparison are listed in Tab. S2. The full table comparing the EggNOG orthologs resolved by individual genomes is available on Figshare (DOI:10.6084/m9.figshare.28495778, https://figshare.com/ndownloader/files/63547101).

The first difference was related to osmolyte and potassium transporters (Fig. 5, category “Osmoregulation”): Kdp potassium transporter genes were present in almost all soil and freshwater genomes, whereas genes encoding subunits of this transporter were found only in very few hypersaline and marine genomes. Although all investigated genomes encoded multiple subunits of the ABC-type glycine/betaine transporter, genomes from hypersaline and salt-saturated mats encoded an additional, alternative, permease component (COG4176 as opposed to universally present COG1174, Fig. 5, category “Osmoregulation”). Additionally, hypersaline mat genomes and some marine genomes encoded a Na^+^-coupled choline–glycine betaine transporter (COG1292) that was absent from freshwater and surface soil genomes (Fig. 5). Genetic repertoires were different not only for osmolyte transporter genes but also for osmolyte synthesis–related genes: most hypersaline genomes encoded trehalose-6-phosphate synthase (COG0380) and glycosylglycerol-phosphate phosphatase (ENOG5028IUG), whereas these genes were absent from surface soil genomes and barely present in freshwater genomes. In contrast, all soil and freshwater genomes encoded the maltoligosyltrehalose synthase (COG3280) compared to 44% of the hypersaline genomes.

The second difference was the much higher frequency of catalase and peroxidase encoding genes in soil, freshwater, and (for some genes) in marine genomes (Fig. 5). Only a minority of salt saturated and hypersaline cyanobacterial genomes encoded such enzymes. Especially striking was the presence of Mn-based catalase genes in all soil-derived cyanobacterial genomes (Fig. 5). Mn ions and Mn-based catalase were already noticed before as potentially having a key role in desiccation survival [75]. Therefore, we searched for the Mn-catalase encoding genes among all public microbial genomes on the JGI genome portal. Indeed, Mn-catalase genes were mainly found in soil microorganisms and were nearly absent from marine genomes (Fig. S5).

The third difference was that the gene encoding sulfide–quinone reductase and thus enabling anoxygenic photosynthesis via oxidation of sulfide was much more common among hypersaline cyanobacterial genomes.

## Discussion

### Differences between high-salinity site and lower-salinity site

Comparing the composition of microbial communities in morphologically identical mats experiencing different salinity regimes, our data indicate that the communities at the HS site largely represent a subset of communities at the LS site. The harsher conditions in terms of higher salinity and likely more frequent periods of salt saturation at the HS site select for a specific subset of microbial species in the microbial mats, offering a few new niches in comparison to the LS site. This is in line with the observation of our previous study, showing that microbial activity mainly happens in periods of salt dilution [24]. A detailed study focusing on intra-species population dynamics of abundant brine-dwelling archaeon *Haloquadratum walsbyi* found a temporal increase in diversity upon experimentally introduced salinity reduction, likely due to decline of the dominant specialist strains and enrichment of more generalist strains [76]. A mesocosm study subjecting microbial mats from a solar saltern to periods of salt dilution and saturation found that different disturbance regimes select for different species within the same genera (“congeneric species replacement”) over the course of over 2 years [23]. Even though we do see some examples of congeneric species selection, e.g. in *Salinigranum* archaea (see relative abundances of MAGs in File S1), most differential abundance patterns play out at higher taxonomic levels and also manifest themselves as differences in functional potential between HS and LS sites (Fig. 2C). These more pronounced differences compared to the controlled salinity shift experiments in solar salterns [23, 76] are almost certainly caused by (i) a longer selection process and (ii) greater differences in tidal settings between HS and LS sites.

MAGs with anaerobic and microaerophilic potential were much more abundant at LS site (especially at 2–3 mm depth) than at HS site (Fig. 2C), despite the LS site having much higher oxygen concentration during the day that can reach deeper into the mat (Fig. 1F). A possible explanation could be provided by energetic constraints. Anaerobic metabolisms generate less energy than aerobic ones. Therefore, anaerobes are left with less energy for countering the saturation-level salinity by osmoregulation. Although a fermentative lifestyle is not expected to be thermodynamically inhibited at any salt concentration [12], higher salinity is still more energetically demanding and could still slow down growth. Although oxidative stress during the day might be lower at the HS site, at night, both mats turn anoxic [24]. The hypersalinity stress at the HS site, however, is higher than at the LS site at any time of the day. This could explain why anaerobic organisms can grow better at lower salinities of LS. At least some of the MAGs with microaerophilic and anaerobic genomic potential contained genes to cope with oxygen stress, likely enabling them to survive the oxic daytime conditions. However, the majority did not and their sensitivity toward oxygen and their potential coping strategies remain unclear.

Another difference in community composition between the two sites was the high relative abundance of eukaryotic sequences, e.g. of diatoms, at the HS site. It is unlikely that diatoms grow better at saturation-level salinities [13, 77] An explanation for their higher proportions at the HS site might be the lack of their decomposition, due to the high cost of osmoregulation at salt saturation and lack of oxygen. It could also be merely a reflection of temporal and spatial variability of diatom occurrence at these sites.

### Attributions of metabolic capabilities to taxa

Although the range of metabolisms occurring in a microbial mat is well known [2, 78], the genomic potential of the resident populations has so far been only characterized in broad metabolic categories present in the community as a whole and for lower-salinity mats such as at Guerrero Negro saline in Mexico [79], Highbourne Cay in the Bahamas [80], Shark Bay in Australia [17, 21, 81], and Araruama lagoon in Brazil [18]. The findings of our genome-resolved investigation, e.g. regarding sulfur-oxidation capability, show that we still cannot predict the function of uncultured microbial mat organisms from their taxonomic identity. Although in other microbial mats *Gammaproteobacteria* are often considered to be major sulfide oxidizers [1, 18, 30], we found S-oxidation potential among *Cyanobacteria*, *Chloroflexota*, and *Rhodovibrio Alphaproteobacteria*. S-oxidation in *Cyanobacteria* and *Chloroflexota* was previously known [38, 40, 43], whereas the two existing isolates of *Rhodovibrio* were considered photoheterotrophic [82–84]. Based on our data, however, *Rhodovibrio* might be one of the major phototrophic (via Sqr) and chemolithotrophic (via the SOX system) sulfide oxidizers in hypersaline microbial mats. The discrepancy between the sulfur-oxidizing potential of the community assumed based on taxonomy and previously studied closest relatives alone and based on actual metagenomic data (illustrated in Fig. S6) is a prime example of why scientists still need to be very cautious when predicting traits based on taxonomic markers alone.

Another genomic trait that could not have been assumed based on taxonomy was the genetic potential for potassium-based osmo-regulation (salt-in strategy) in *Myxococcota*, specifically the *Bradymonadaceae* family. So far *Bradymonadaceae* are represented by cultures of marine predatory bacteria [85, 86] with a preference for *Bacteroidota* as prey [85]. The *Salinibacteraceae* family, first bacteria that was shown to use the salt-in osmoregulation strategy [11], used to be considered part of *Bacteroidota* phylum (now *Rhodothermota* [87]). If *Bradymonadaceae* in the studied mats prey on *Salinibacteraceae*, they might have acquired the potassium transporter genes from their potential prey via horizontal gene transfer. Our phylogenetic analysis of the potassium transporter genes showed that TrkA potassium transporters encoded by the *Bradymonadaceae* MAGs were indeed closely related to *Salinibacter* and *Archaea* TrkA (Fig. S3), whereas TrkH were not (Fig. S4). Although it seems certain that halophilic *Bradymonadaceae* use the salt-in osmoregulation strategy, its evolutionary origin might be more complex than a single horizontal transfer of the entire gene set and requires further investigation.

The salt-in osmoregulation strategy, combined with an acidic proteome, is mostly found in extremely halophilic organisms [9]. Earlier bulk metagenomic studies of microbial mats suggested an evolutionary convergence toward an acidic proteome among all members of hypersaline microbial mat communities [79], whereas later evaluation of isolate genomes from different environments questioned such uniformity [88]. With our isolation-independent genome-resolved metagenomics approach, we show a binomial distribution of average isoelectric points of proteins among the community members (Fig. 4). It also showed that microorganisms with a neutrophilic proteome and salt-out osmoregulation such as *Rhodovibrio* sp. were able to thrive in extremely hypersaline environments. However, it remains to be tested if the opposite is also valid: can microorganisms with a salt-in osmoregulation strategy be found thriving in low salinity environments? *Bradymonadaceae* MAGs, for example, were more abundant in LS site mats, despite having the genes for the salt-in strategy and a very acidic proteome. Furthermore, proteins encoded in the genome of the cultured relative *Bradymonas sediminis*, isolated from marine sediment [86], exhibited an average isoelectric point of six, comparable to the one of *Salinibacter* MAGs (Fig. 4), and encoded the necessary potassium transporters (Fig. S3 and S4). However, it is still significantly higher than the isoelectric points of proteins encoded in the *Bradymonadaceae* MAGs obtained in this study (5.3 on average).

Taken together, our findings extend knowledge of metabolisms of halophilic bacteria and improve future predictions of microbial ecological functions and possible microbially mediated processes in hypersaline environments based on microbial community composition.

### Survival strategies of *Cyanobacteria* in water-limited environments

Oxygenic phototrophic microorganisms like *Cyanobacteria* are the only true primary producers in microbial mats. However, their photosynthetic and oxygen producing activity can be inhibited when salinity approaches saturation [16, 24, 89]. One could assume that in these periods, other microorganisms like anoxygenic phototrophic *Proteobacteria* would take on the role of primary producers. However, our analysis of cyanobacterial population genomes indicated that switching to anoxygenic photosynthesis based on phototrophic sulfide oxidation might enable cyanobacteria to gain energy in periods when oxygenic photosynthesis is not possible. By being able to generate energy to sustain their cells, sulfur-oxidizing cyanobacteria would not need to enter a dormant state to survive high salinities. Even though protomotive force, and thus ATP, can also be generated via cyclic electron transfer around photosystem I, cyclic electron transfer does not generate reduction equivalents needed for the CBB cycle. In contrast, phototrophic sulfide oxidation could power CO_2_ fixation and growth so that *Cyanobacteria* would remain the main primary producers, even at salt saturated conditions. This is consistent with our previous measurements of active light-dependent sulfide oxidation even at saturation-level salinities and our microscopic observations of intact cyanobacteria filaments with no visible resting-stage (e.g. akinetes) formation [24]. Cyanobacteria isolated from various environments are known to be capable of sulfide oxidation [39, 40, 42]. In the studied microbial mats, every cyanobacterial population was found to encode sulfur-oxidation genes (Fig. 3), indicating a wide distribution and thus importance of this metabolism in a hypersaline environment. The widespread sulfur oxidation potential among cyanobacteria aligns well with the assumption that hypersaline microbial mats might represent modern day analogues of microbial communities of the early Earth, where ancient *Cyanobacteria* performed anoxygenic photosynthesis before oxygenic photosynthesis evolved [90].

Energy provided by the phototrophic sulfide oxidation enables cyanobacteria to counter osmotic stress and retain a necessary amount of water in the cytoplasm. We found genes for osmoregulation via small organic molecules like glycine–betaine in most cyanobacterial MAGs obtained in this study (Fig. 5). Osmotic stress has been compared to desiccation stress in the past [7]. However, when comparing genomes of filamentous cyanobacteria from soils, freshwater, and marine habitats to the ones from hypersaline and salt-saturated environments, we identified clear differences in osmoregulation-related genes, with hyperhalophile genomes encoding additional, different glycine–betaine transporters and different pathways of trehalose synthesis (Fig. 5). Genomes of freshwater cyanobacteria were highly similar to those of soil cyanobacteria with respect to genes involved in osmoregulation and ROS stress resistance. The freshwater genomes used in this comparison originated from small springs, which are likely to at least partially dry out in summer and therefore also represent a desiccation-prone environment (Tab. S3). More elaborate osmoregulation in genomes of halophilic cyanobacteria as compared to surface soil or fresh water ones might be owed to the fact that water is generally present in their native environment but needs to be retained in the cells or even “drawn” into the cell against the high osmotic pressure created by the high salinity. During desiccation, water is completely absent and the cell needs to be preserved to stay intact in an anhydrobiotic state [91]. Further differences between desiccation-tolerant and halophilic cyanobacteria genomes were related to ROS defense and the capability of sulfide oxidation. Unlike halophilic cyanobacteria, their terrestrial relatives, even from the same family as in case of *Coleofasciculaceae*, do not have the genetic potential for sulfide oxidation. At the same time, halophilic cyanobacteria seem to often lack catalase genes, especially Mn-based catalase, needed for neutralizing ROS. The likely connection between these two differences is that the sulfidic environment arising during saturation–salinity periods due to inhibition of oxygenic photosynthesis but ongoing respiration (i) eliminates the need for ROS defense because oxygen is low or absent and (ii) offers the possibility of energy generation from phototrophic oxidation of sulfide. Conversely, in dry soil, oxygen is abundant and sulfide is absent, which might explain why only few of the analyzed terrestrial cyanobacteria genomes encode sulfur-oxidation genes but most encode additional ROS-defense mechanisms. Specifically, we identified the Mn-based catalase [92] as a general key ROS-defense gene in terrestrial cyanobacterial genomes. Its generally high frequency among bacterial and archaeal genomes from terrestrial air-exposed environments, lower frequency in hypersaline/salt-saturated habitats, and its near absence from planktonic or benthic marine environments (Fig. S5) calls for more in-depth investigation of its function and importance during desiccation survival. Supporting its critical role during desiccation, a recent study has found that Mn-based catalase genes are expressed by biological soil crust microbiota during desiccation as opposed to Fe-based catalases that are expressed in the hydrated state [75]. The function of Mn^2+^-ions as effective antioxidants is known from radiation- and desiccation-tolerant microorganisms [93–95]. However, the specific role of the Mn-based catalase for desiccation tolerance is yet to be elucidated.

Taken together, our findings highlight very significant differences in microbial adaptation to desiccation as opposed to hypersalinity and should serve as a caution for future studies not to generalize and equate these two environmental settings.

## Conclusions

Besides revealing metabolisms previously not known to exist in certain halophile taxa such as S-oxidation in *Rhodovirbiro* and salt-in osmoregulation in *Bradymonadaceae*, our study contributes to a better understanding of microbial adaptations to stresses imposed by hypersalinity and desiccation. Building on the first explorative study of microbial communities of the Shannah sabkha [15] and a second process-oriented investigation of these microbial mats [24], we were able to find two locations in the sabkha with morphologically identical microbial mats experiencing different salinity regimes. Using this “natural salinity shift experiment” for comparison of microbial taxa and their functional potential selected for by different salinity levels, we came to the conclusion that saturation-level salinity mainly selects for a narrower subset of taxa from the community found at lower salinity rather than promoting growth of a distinct microbiome. Another comparison that we were able to make is between genomes of filamentous cyanobacteria from terrestrial, aquatic, and hypersaline habitats. Their genetic potentials reflect the fundamental differences between hypersalinity stress in an aquatic environment and desiccation stress in a terrestrial environment (Tab. 1): (i) In a hypersaline/salt-saturated environment, water can be retained or drawn into the cell if energy for osmoregulation is available, whereas during desiccation, water is physically absent and the cell needs to be prepared for anhydrobiosis. (ii) A desiccated terrestrial environment has full oxygenation and thus high oxygen stress whereas in hypersaline conditions oxygen levels are decreased by reduced oxygen solubility, inhibited photosynthesis combined with ongoing respiration and, in marine environments, oxygen scavenging by sulfide due to ongoing sulfate reduction. Thus, desiccation tolerant cyanobacteria show genetic signatures of adaptations to ROS stress and cell preservation in absence of water, whereas halophilic cyanobacteria genomes indicate the ability to switch from oxygenic photosynthesis to phototrophic S-oxidation to keep producing energy for growth and osmoregulation.

**Table 1:** Differences in challenges to microbial metabolism imposed by desiccation and hypersalinity.

	**Desiccation (e.g. in dry surface soils)**	**Hypersalinity in microbial mats and sediments**
**Availability of water**	Low or not available due to evaporation after rainfall.	Low availability through hyperosmotic condition, but the mat/sediment matrix is water-saturated.
**Presence of oxygen**	Gas exchange is possible due to air-filled soil pores. Oxygen can be present at nearly atmospheric levels.	Solubility of oxygen in salt-saturated water is low, oxygen production by photosynthesis is inhibited, while aerobic respiration is still active consuming the remaining oxygen.
**Intensity of ROS stress**	Abundance of oxygen in combination with Fenton reactions during desiccation of cells and other factors like UV lead to high amounts of ROS	Less oxygen results in lower amounts of ROS. Higher amounts of sulfide act as additional oxygen scavengers.
**Metabolic activity**	Extremely reduced at low water availability or halted below minimum threshold	Possible if osmotic pressure is countered
**Cellular response**	Cell preservation by vitrification, accumulation of ROS scavengers, formation of resting cell forms, preparation for later resuscitation, and repair once rehydrated	Osmoregulation, switch to alternative energy generation (e.g. heterotrophy, cyclic electron transfer, phototrophic sulfide oxidation)

## Supplementary Material

ISMEJ_D_25_02479R1_Supplementary_material_revised_wrag166

File_S1_Functions_per_MAG_revised_wrag166

## Data Availability

The metagenomic sequencing, assembly, and annotation was performed by the Joint Genome Institute (Department of Energy, USA) under the Community Sequence Program (Proposal ID: 504992 https://doi.org/10.46936/10.25585/60000995). All information and data can be accessed under the GOLD study ID Gs0144445 through the JGI Genome portal or IMG-M/ER interface. Raw sequence data is deposited in the INSDC databases under the accession numbers: PRJNA617109, PRJNA653441-PRJNA653450, PRJNA653497-PRJNA653512, PRJNA677340, and PRJNA677341. Code documenting the exact analysis parameters (https://doi.org/10.6084/m9.figshare.28494914), tables with analysis results (https://doi.org/10.6084/m9.figshare.28495778), and metagenome-assembled genomes (https://doi.org/10.6084/m9.figshare.28494731) are deposited on Figshare under project number 238424.
