# Supplementary material for: Sulfide-oxidizing potential and hypersalinity tolerance strategies in salt-crust covered coastal microbial mats
Source: ISME J. 2026 Jun 26;20(1):wrag166. doi: 10.1093/ismejo/wrag166 (PMC13384644; doi:10.1093/ismejo/wrag166)
Supplement: ISMEJ_D_25_02479R1_Supplementary_material_revised_wrag166 [file ismej_d_25_02479r1_supplementary_material_revised_wrag166.pdf]

Sulfide-oxidizing potential and hypersalinity tolerance strategies in salt-crust covered coastal microbial mats - Supplementary Material

Dimitri V. Meier[1,2], Andreas Greve[3], Dirk de Beer[3], Raeid M. M. Abed[4], Dagmar Woebken[2]

[1]*Chair of Ecological Microbiology, Bayreuth Center of Ecology and Environmental Research (BayCEER), University of Bayreuth, Germany*

[2]*Department of Microbiology and Ecosystem Science, Centre for Microbiology and Environmental Systems Science, University of Vienna, Vienna, Austria*

[3]*Max Planck Institute for Marine Microbiology, Bremen, Germany*

[4]*Biology Department, College of Science, Sultan Qaboos University, Muscat, Sultanate of Oman*

Figure S1

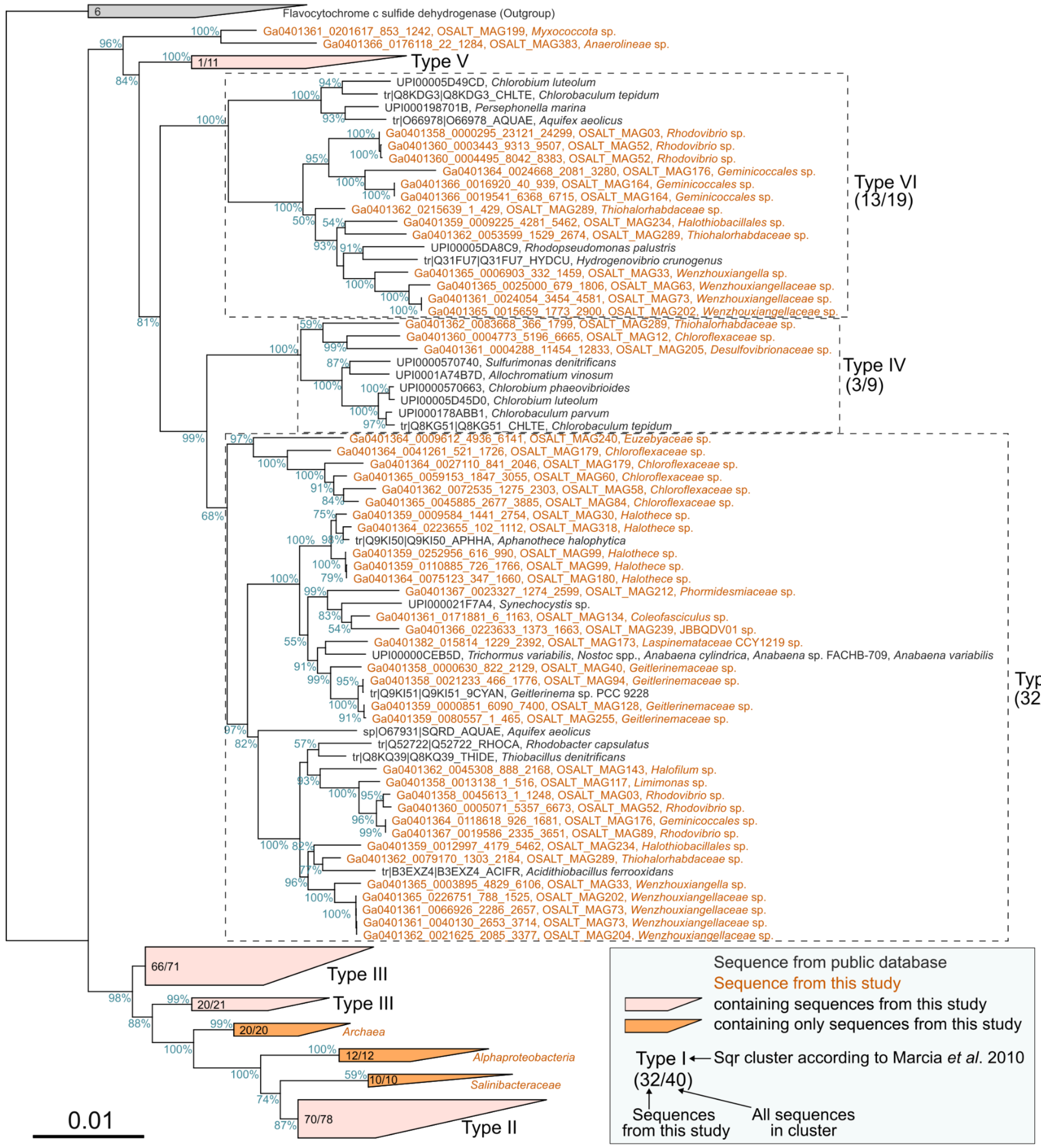

Figure S1: Phylogenetic tree of Sulfide:quinone reductase (Sqr) amino acid sequences containing reference sequences obtained from NCBI Protein database and Uniprot and Sqr sequences from the MAGs generated in this study. The clusters were named based on the placement of reference sequences in the tree by Macia et al. 2010[1]. The clusters I, IV, and VI containing known sulfide-oxidizing chemolithotrophic microorganisms are expanded for detailed view. Sqr amino acid sequences were aligned using MAFFT v. 7.490 [2] in L-INS-I mode, and used for phylogenetic tree calculations with FastTree v. 2.1.11 [3] based on alignment positions conserved in at least 25% of the sequences. The tree was calculated with the Le-Gascuel substitution model [4], BioNJ [5] starting tree and gamma likelihood optimization. Tree visualization and exploration was done in ARB v. 7.0 [6]. Full tree is available on Figshare under DOI:10.6084/m9.figshare.28494914 (see 'Analysis_and_plotting')

Figure S2

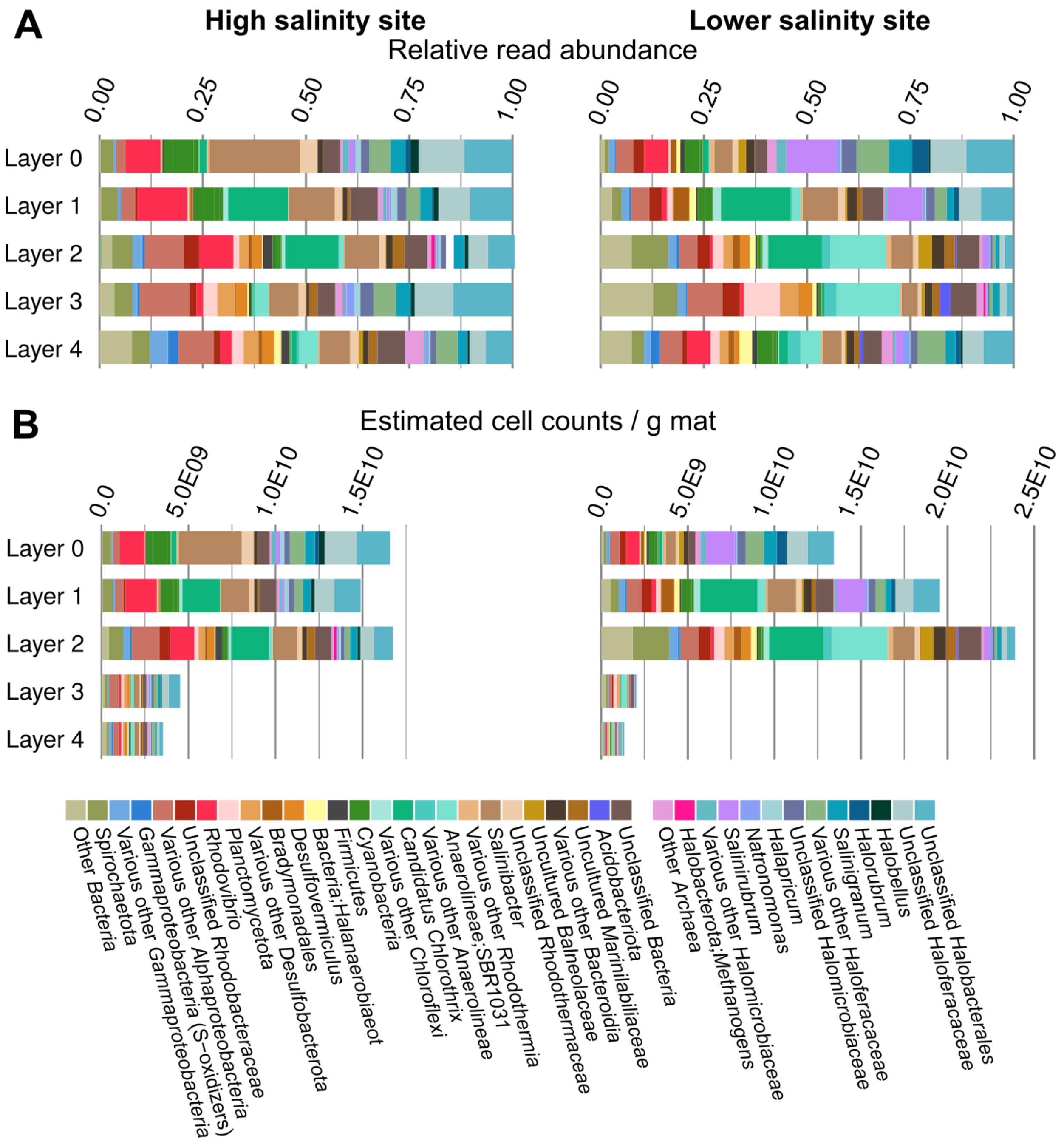

Figure S2: Taxonomic composition of the sequenced metagenomes based on mapping of unassembled metagenomic reads to SILVA SSU database (v. 138.1) with PhyloFlash. The bar charts show average relative abundances based on three replicates per mat layer. (A) Unscaled relative read abundances. (B) Abundances scaled by total cell counts obtained by fluorescence microscopy.

Figure S3

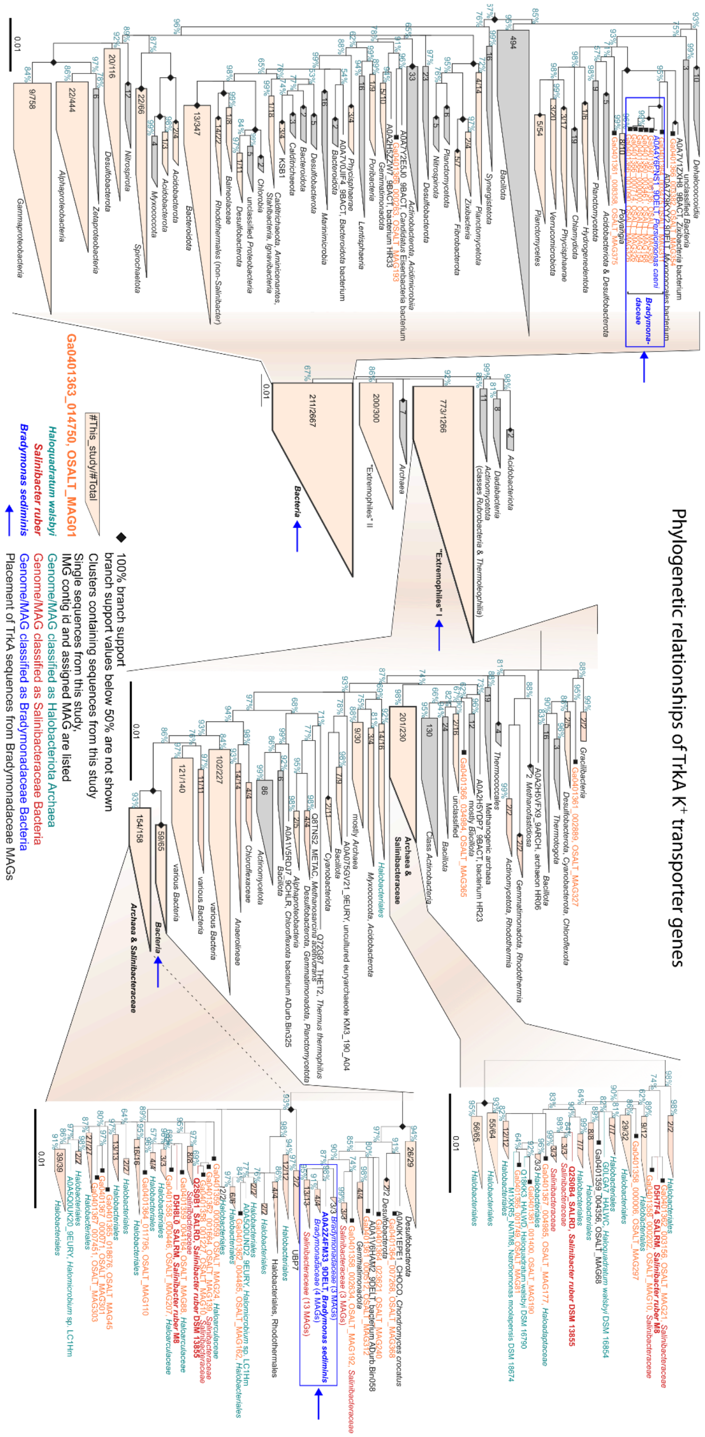

Figure S3: Phylogenetic tree of TrkA potassium transporter genes involved in "salt-in" hypersalinity tolerance strategy in archaea and bacteria. The tree was calculated based on a MAFFT alignment [2] with a 25% positional conservation filter using FastTree2 [3] with Le-Gascuel substitution model [4], BioNJ [5] starting tree and gamma likelihood optimization.

Figure S4

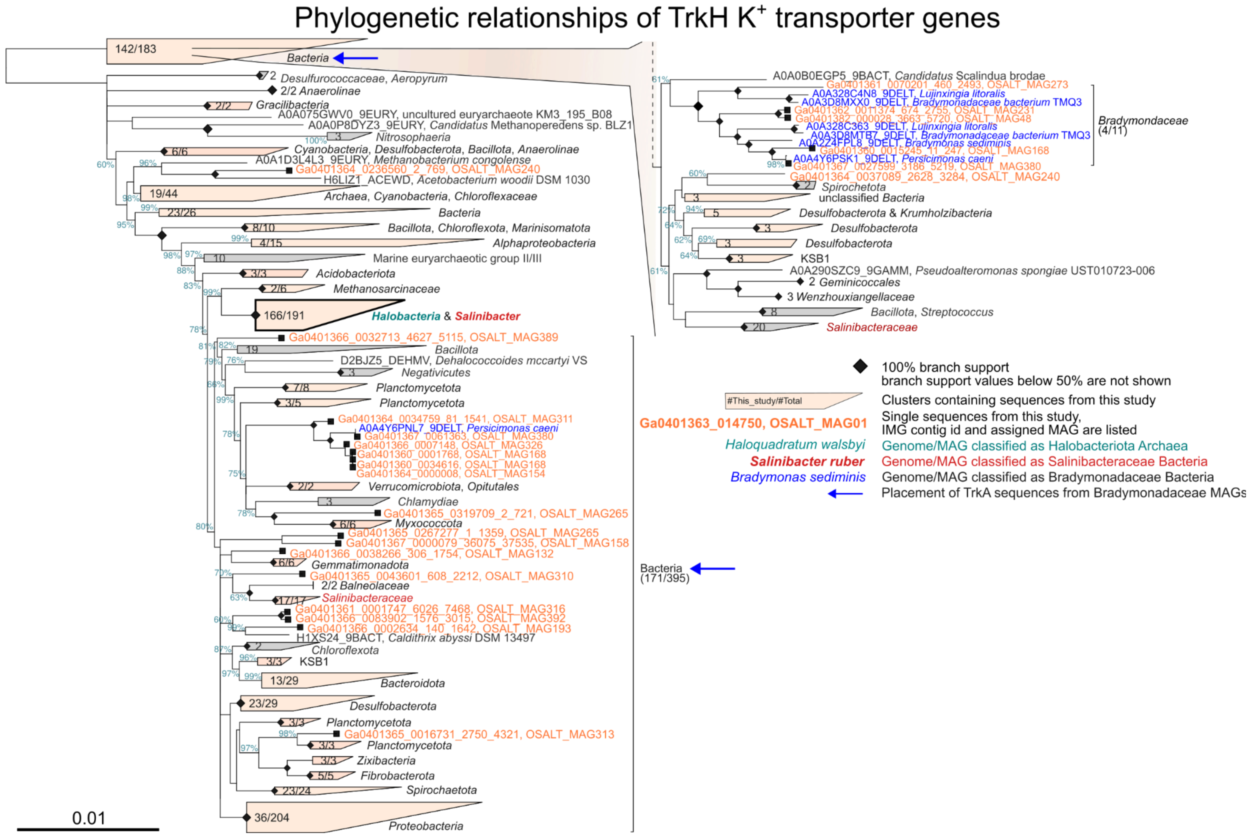

Figure S4: Phylogenetic tree of TrkH potassium transporter genes involved in “salt-in” hypersalinity tolerance strategy in archaea and bacteria. The tree was calculated based on a MAFFT alignment [2] with a 25% positional conservation filter using FastTree2 [3] with Le-Gascuel substitution model [4], BioNJ [5] starting tree and gamma likelihood optimization.

Figure S5

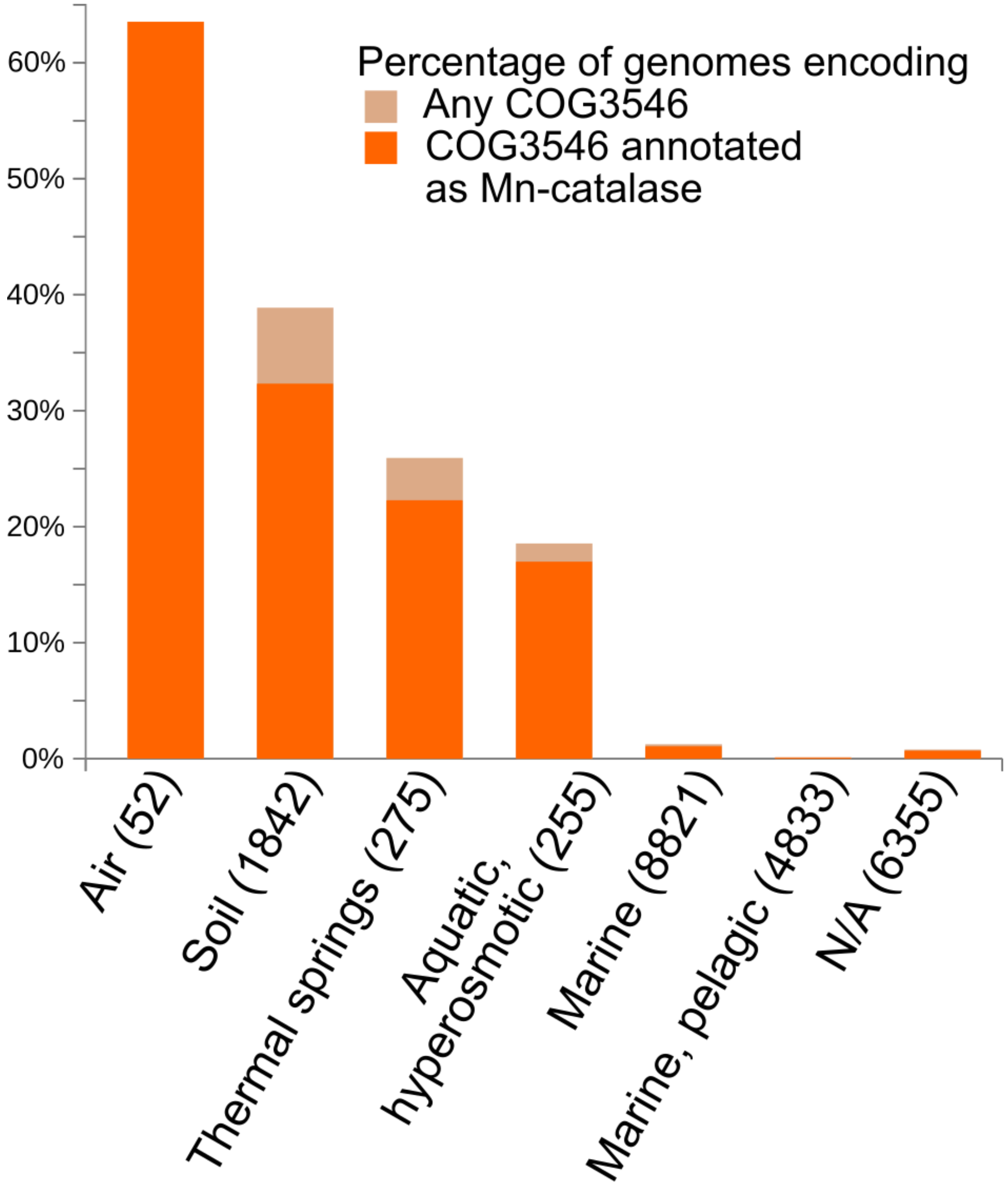

Figure S5: Distribution of Mn-based catalase among the public microbial genomes deposited at the JGI genome portal (accessed on 02.04.2026). The bars show the percentages of genomes from a given habitat category that encode a protein from the COG03546 orthologous group. Numbers in brackets indicate the number of genomes in each of the categories. IMG annotates COG03546 proteins as either ‘manganese catalase’, ‘spore coat protein JC’ or ‘bacterioferritin’. While the search was limited to bacterial and archaeal genomes with an assigned ecosystem category “Air”,”Aquatic” or “Terrestrial”, it also returned genomes without any assigned ecosystem category which are depicted here as “N/A”.

Figure S6

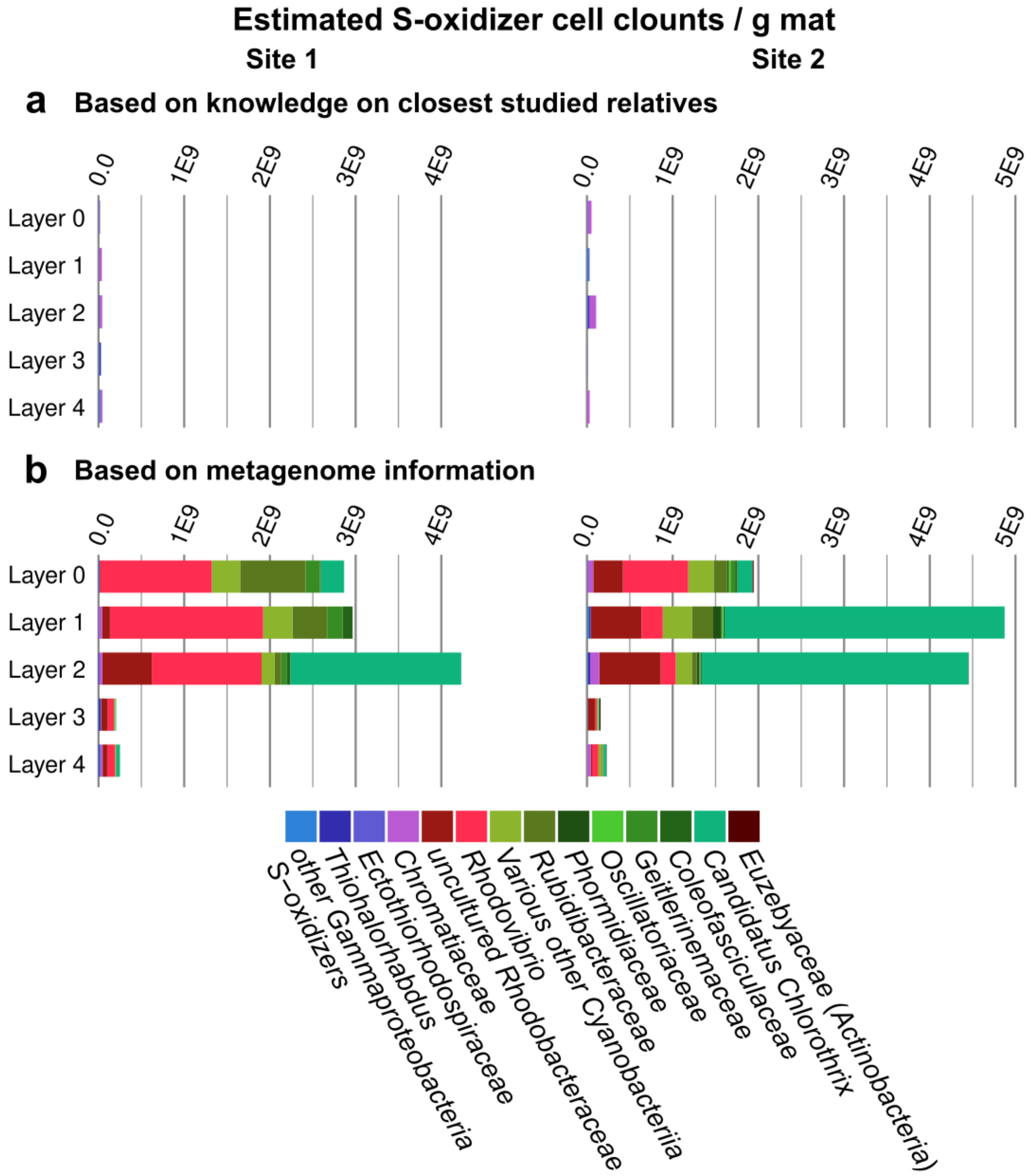

Figure S6: Estimated abundance and taxonomic composition of sulfur-oxidizing bacteria in the studied microbial communities based on metagenomic SSU rRNA data (Fig. S1) and functional assignment based on taxonomy. Panel **a** shows estimated S-oxidizer abundance solely based on previous knowledge about closest relatives of detected microbial taxa. Panel **b** shows estimated abundances of S-oxidizers based on sulfur oxidation genes detected in related MAGs assembled from the metagenomes.

Table S1: Metagenomic dataset metrics

Sample	EBI/NCBI project id	JGI project Id	Reads	Percentage mapped to MAGs
Site 1 Mat II Layer 0	PRJNA653507	1251999	37,280,248	50%
Site 1 Mat II Layer 1	PRJNA653508	1252001	31,967,800	49%
Site 1 Mat II Layer 2	PRJNA653509	1252003	38,237,262	52%
Site 1 Mat II Layer 3	PRJNA653510	1252005	303,324,610	37%
Site 1 Mat II Layer 4	PRJNA677340	1252007	36,813,099	24%
Site 1 Mat III Layer 0	PRJNA653511	1252009	45,868,310	53%
Site 1 Mat III Layer 1	PRJNA653512	1252011	52,409,494	51%
Site 1 Mat III Layer 2	PRJNA653441	1252013	67,526,582	29%
Site 1 Mat III Layer 3	PRJNA653442	1252015	41,192,110	46%
Site 1 Mat III Layer 4	PRJNA677341	1252017	31,579,823	29%
Site 1 Mat V Layer 0	PRJNA653497	1251979	276,138,580	44%
Site 1 Mat V Layer 1	PRJNA653498	1251981	290,660,276	45%
Site 1 Mat V Layer 2	PRJNA653499	1251983	130,676,270	43%
Site 1 Mat V Layer 3	PRJNA653500	1251985	171,588,514	37%
Site 1 Mat V Layer 4	PRJNA653501	1251987	154,097,556	32%
Site 2 Mat IX Layer 0	PRJNA653502	1251989	89,991,372	40%
Site 2 Mat IX Layer 1	PRJNA653503	1251991	223,149,260	41%
Site 2 Mat IX Layer 2	PRJNA653504	1251993	253,149,770	38%
Site 2 Mat IX Layer 3	PRJNA653505	1251995	287,532,180	30%
Site 2 Mat IX Layer 4	PRJNA653506	1251997	199,560,972	27%
Site 2 Mat VII Layer 0	PRJNA653443	1252019	44,003,602	30%
Site 2 Mat VII Layer 1	PRJNA653444	1252021	50,219,208	31%

Sample	EBI/NCBI project id	JGI project Id	Reads	Percentage mapped to MAGs
Site 2 Mat VII Layer 2	PRJNA617109	1252023	34,972,892	34%
Site 2 Mat VII Layer 3	PRJNA653445	1252025	44,232,380	41%
Site 2 Mat VII Layer 4	PRJNA653446	1252027	54,976,364	45%
Site 2 Mat VIII Layer 0	PRJNA653447	1252029	30,333,978	46%
Site 2 Mat VIII Layer 1	PRJNA653448	1252031	32,738,352	45%
Site 2 Mat VIII Layer 2	PRJNA653449	1252033	57,422,358	49%
Site 2 Mat VIII Layer 3	PRJNA653450	1252035	49,494,372	37%

Table S2: Genetic indicators of predicted MAG metabolisms

Predicted metabolism	Indicator gene(s)
**CO_2-fixing (CBB cycle)**	RuBisCO (Type I)
**CO_2-fixing (3−hydroxypropanoate cycle)**	Biotin carboxylase + Malonyl-CoA reductase + Propionyl-CoA_synthase
**CO_2-fixing (reverse TCA cycle)**	ATP-citrate lyase
**CO_2-fixing (reverse Acetyl−CoA pathway)**	Acetyl-CoA_synthase/CO-dehydrogenase (anaerobic)
**N_2−fixing**	NifH
**Phototrophic (chlorophyll, Cyanobacteria)**	Photosystem I (>= 3 different genes) + Photosystem II (>= 10 different genes)
**Phototrophic (bacteriochlorophyll)**	>= 3 different chlorophyll synthesis or anoxygenic photosystem genes
**Photoheterotrophic (bacteriorhodopsins)**	Rhodopsin-encodin genes
**O2-respiring**	Cytochrome c oxidase | cytochrome bd oxidase
**NO_3^-−reducing**	Dissimilatory nitrate reductase (nap, nar)
**NO_2^-−reducing**	Dissimilatory nitrite reductase (nir, nrf)
**NO-reducers**	Nitric oxide reductase (nor)
**N_2O-reducers**	Nitrous oxide reductase (nos)
**SO_4^{2-}−reducing**	Dissimilatory sulfate reductase (The two subunits encoded separately as dsrA and dsrB, not a fused dsrAB)
**Anaerobic & microaerophilic**	No genes of cytochrome c oxidases, if cytchrome bd oxidase genes → microaerophiles
**Fermenting**	No respiratory genes: Cyt c oxidase, Cyt bd oxidase, Dissimilatory N-reduction, Sulfate reduction, or methanogenesis
**Multi−heme cytochromes**	Multi-heme cytochromes lacking more specific functional annotations
**Methanogenic**	Methyl-Coenzyme M reductase subunit A (mcrA)
**H_2S−oxidizers**	Sulfide:quinone reductase Type I, IV or VI | > 1 gene of the SOX system)

Table S3: Genomes used for comparative genomic analysis of *Cyanobacteria* (Fig. 5)

Accession	Organism name	GTDB taxonomy (Family, Genus)	Habitat category	Source
GCA_003511885.1	*Limnofasciculus sp003511885*	*Coleofasciculaceae; Limnofasciculus*	Fresh-water	Urban waterways Industrial post rain sediment;Singapore
GCA_003695795.1	*DVED01 sp003695795*	*Geitlerinemataceae; DVED01*	Fresh-water	iron-rich hot spring;Japan: Tokyo
GCA_003542015.1	*Microcoleus sp003542015*	*Microcoleaceae;Microcoleus*	Fresh-water	microbial mat
GCA_004292745.1	*Microcoleus anatoxicus*	*Microcoleaceae;Microcoleus*	Fresh-water	river bed microbial mat;USA: California, Eel River, Larabee Creek near mouth
GCA_004292955.1	*Microcoleus sp004292955*	*Microcoleaceae;Microcoleus*	Fresh-water	river bed microbial mat;USA: California, Eel River, Middle Fork Eel River
GCA_004293365.1	*Microcoleus sp004293365*	*Microcoleaceae;Microcoleus*	Fresh-water	river bed microbial mat;USA: California, Eel River, South Fork Eel River near Piercy
GCA_004294385.1	*Microcoleus sp004294385*	*Microcoleaceae;Microcoleus*	Fresh-water	river bed microbial mat;USA: California, Eel River, Elder Creek
GCA_013299165.1	*Microcoleus sp013299165*	*Microcoleaceae;Microcoleus*	Fresh-water	biofilm;New Zealand: Wai-iti river, Nelson
GCA_013299185.1	*Microcoleus sp013299185*	*Microcoleaceae;Microcoleus*	Fresh-water	biofilm;New Zealand: Wai-iti river, Nelson
GCF_002412335.2	*Microcoleus bourrellyi*	*Microcoleaceae;Microcoleus*	Fresh-water	lake water;Italy: Lake Garda
GCF_003003725.1	Microcoleus sp003003725	Microcoleaceae;Microcoleus	Fresh-water	freshwater lake;Canada: Quttinirpaaq Lagoon
GCA_011390515.1	*Laspinema sp011390515*	*Laspinemataceae; Laspinema*	Fresh-water	lacustrine sediment;China: Xiaochaidan Lake
GCA_010671925.1	*Moorena sp010671925*	*Coleofasciculaceae; Moorena*	marine	marine benthic turfs;American Samoa
GCA_010692325.1	*Moorena sp010692325*	*Coleofasciculaceae; Moorena*	marine	marine benthic turfs;Papua New Guinea: Hoia Bay
GCA_010692395.1	*Moorena sp010692395*	*Coleofasciculaceae; Moorena*	marine	marine benthic turfs;Papua New Guinea: Hoia Bay

Accession	Organism name	GTDB taxonomy (Family, Genus)	Habitat category	Source
GCA_011524395.1	*Moorena sp011524395*	*Coleofasciculaceae; Moorena*	marine	coral reef macroalgae biofilm;Australia, Great Barrier Reef
GCF_000211815.1	*Moorena producens*	*Coleofasciculaceae; Moorena*	marine	coral rubble and rocks at 2-3 m depth;Netherlands Antilles, Curacao
GCF_001767235.1	*Moorena producens_A*	*Coleofasciculaceae; Moorena*	marine	shallow tropical waters; normally from coral reef substrate;Pacific Ocean: Palmyra Atoll
GCF_001942495.1	*Moorena bouillonii*	*Coleofasciculaceae; Moorena*	marine	coral reefs at a depth of 10 m;Papua New Guinea, New Ireland
GCA_010672925.1	*Caldora sp010672925*	*Coleofasciculaceae; Caldora*	marine	marine benthic turfs;American Samoa
GCA_010692635.1	*Caldora sp010692635*	*Coleofasciculaceae; Caldora*	marine	marine benthic turfs;Panama: Boco del Toro
GCA_010692425.1	*SIO3C6 sp010692425*	*Coleofasciculaceae; SIO3C6*	marine	marine benthic turfs;Panama: Portobelo
GCA_010692645.1	*SIO3C6 sp010692645*	*Coleofasciculaceae; SIO3C6*	marine	marine benthic turfs;Papua New Guinea: Hoia Bay
GCF_012295525.1	*Oxynema aestuarii*	*Laspinemataceae; Oxynema*	marine	mangrove intertidal soil surface;none
GCF_000473895.1	*Rubidibacter lacunae*	*Rubidibacteraceae; Rubidibacter*	marine	sea water;Micronesia, Chuuk lagoon
GCA_010672865.1	*IIICB1 sp010672865*	*SIO1D9; IIICB1*	marine	marine benthic turfs;China
GCF_000155555.1	*Coleofasciculus chthonoplastes_A*	*Coleofasciculaceae; Coleofasciculus*	hypersaline	salt marsh;USA, Woods Hole
GCA_012034575.1	*Microcoleus sp012034575*	*Microcoleaceae; Microcoleus*	hypersaline	stromatolite mat, marine tidal flow zone;South Africa, Cape Recife
GCA_012035135.1	*Microcoleus sp012035135*	*Microcoleaceae; Microcoleus*	hypersaline	stromatolite mat, marine tidal flow zone;South Africa, Cape Recife
GCA_009909085.1	*Halothece sp009909085*	*Rubidibacteraceae;Halothece*	hypersaline	microbial mat;USA: Bridger Bay in Great Salt Lake, Utah
GCF_000317615.1	*Halothece salina*	*Rubidibacteraceae;Halothece*	hypersaline	solar lake;Israel

Accession	Organism name	GTDB taxonomy (Family, Genus)	Habitat category	Source
GCF_000317635.1	*Halothece sp000317635*	*Rubidibacteraceae;Halothece*	hypersaline	Water; lake; Solar lake;Israel: Solar lake
GCF_007904085.1	*Halothece natronophila*	*Rubidibacteraceae;Halothece*	hypersaline	soda lake Magadi;Kenya: Eastern African Rift Valley
GCA_012031635.1	*JAAUUE01 sp012031635*	JAAUUE01;JAAUUE01	hypersaline	stromatolite mat, marine tidal flow zone;South Africa:Schoenmakerskop
GCF_001870905.1	*PCC-9228 sp001870905*	*Geitlerinemataceae_A; PCC-9228*	hypersaline	water;Israel: Solar Lake
GCA_003021505.1	*QS-4-48-99 sp003021505*	*Rubidibacteraceae; QS-4-48-99*	salt_crust	desert salt crust;Chile: Atacama Desert
GCA_003022125.1	*QS-8-64-29 sp003022125*	*Rubidibacteraceae; QS-8-64-29*	salt_crust	desert salt crust;Chile: Atacama Desert
GCA_003021905.1	*Halothece sp003021905*	*Rubidibacteraceae;Halothece*	salt_crust	desert salt crust;Chile: Atacama Desert
AVCR-Coleofasciculus01	*CADCTM01 sp902805485*	*Coleofasciculaceae; CADCTM01*	soil	soil crust;Israel, Negev Desert, Avdat LTER
GCF_000317515.1	*Allocoleopsis franciscana*	*Coleofasciculaceae; Allocoleopsis*	soil	orchid house soil;USA: San Francisco; CA
AVCR-Microcoleus 01	*Microcoleus AVDCRST_MAG84*	*Microcoleaceae;Microcoleus*	soil	soil crust;Israel, Negev Desert, Avdat LTER
GCF_000214075.1	*Microcoleus vaginatus_A*	*Microcoleaceae;Microcoleus*	soil	desert soil crust;USA
GCF_000317475.1	*Microcoleus sp000317475*	*Microcoleaceae;Microcoleus*	soil	soil in orchid house;USA
GCF_013179805.1	*Microcoleus asticus*	*Microcoleaceae;Microcoleus*	soil	soil?;Portugal, Lisbon
GCF_000317105.1	*Laspinema acuminata*	*Laspinemataceae; Laspinema*	soil	soil;USA
OSALT_MAG82	*Coleofasciculus* sp040220555 (ANI 98.8%, AF 81%)	*Coleofasciculaceae;Coleofasciculus*	hypersaline	Shannah lagoon, Oman
OSALT_MAG40		*Geitlerinemaceae*	hypersaline	Shannah lagoon, Oman
OSALT_MAG173	CCY1219 sp035615515 (ANI 97.4%, AF 82%)	*Laspinemataceae;CCY1219*	hypersaline	Shannah lagoon, Oman

Accession	Organism name	GTDB taxonomy (Family, Genus)	Habitat category	Source
OSALT_MAG180		*Rubidibacteraceae;Halothece*	hypersaline	Shannah lagoon, Oman
OSALT_MAG318		*Rubidibacteraceae;Halothece*	hypersaline	Shannah lagoon, Oman
OSALT_MAG99		*Rubidibacteraceae;Halothece*	hypersaline	Shannah lagoon, Oman
OSALT_MAG239		*SIO1D9;JBBQDV01*	hypersaline	Shannah lagoon, Oman
OSALT_MAG134		*Coleofasciculaceae;*	salt_crust	Shannah lagoon, Oman
OSALT_MAG256		*Coleofasciculaceae;*	salt_crust	Shannah lagoon, Oman
OSALT_MAG128		*Geitlerinemaceae;*PCC-9228	salt_crust	Shannah lagoon, Oman
OSALT_MAG255		*Geitlerinemaceae;*PCC-9228	salt_crust	Shannah lagoon, Oman
OSALT_MAG94	*Geitlerinema* sp. PCC 9228 (ANI 99.7%, AF 95%)	*Geitlerinemaceae;*PCC-9228	salt_crust	Shannah lagoon, Oman
OSALT_MAG30		*Rubidibacteraceae;Halothece*	salt_crust	Shannah lagoon, Oman

References:

1. Marcia M, Ermler U, Peng G, Michel H. A new structure-based classification of sulfide:quinone oxidoreductases. *Proteins* 2010; **78**: 1073–1083.
2. Katoh K, Standley DM. MAFFT multiple sequence alignment software version 7: improvements in performance and usability. *Mol Biol Evol* 2013; **30**: 772–780.
3. Price MN, Dehal PS, Arkin AP. FastTree 2 - approximately maximum-likelihood trees for large alignments. *PLoS One* 2010; **5**: e9490.
4. Le SQ, Gascuel O. An improved general amino acid replacement matrix. *Mol Biol Evol* 2008.
5. Gascuel O. BIONJ: an improved version of the NJ algorithm based on a simple model of sequence data. *Mol Biol Evol* 1997; **14**: 685–695.
6. Ludwig W, Strunk O, Westram R, Richter L, Meier H, Yadhukumar, et al. ARB: a software environment for sequence data. *Nucleic Acids Res* 2004; **32**: 1363–1371.
